# Retention in HIV care during the 3 years following release from incarceration: A cohort study

**DOI:** 10.1371/journal.pmed.1002667

**Published:** 2018-10-09

**Authors:** Kelsey B. Loeliger, Jaimie P. Meyer, Mayur M. Desai, Maria M. Ciarleglio, Colleen Gallagher, Frederick L. Altice

**Affiliations:** 1 AIDS Program, Section of Infectious Diseases, Yale School of Medicine, New Haven, Connecticut, United States of America; 2 Department of Epidemiology of Microbial Diseases, Yale School of Public Health, New Haven, Connecticut, United States of America; 3 Department of Chronic Disease Epidemiology, Yale School of Public Health, New Haven, Connecticut, United States of America; 4 Department of Biostatistics, Yale School of Public Health, New Haven, Connecticut, United States of America; 5 Health and Addiction Services Quality Improvement Program, Connecticut Department of Correction, Wethersfield, Connecticut, United States of America; 6 Centre of Excellence in Research in AIDS, University of Malaya, Kuala Lumpur, Malaysia; Massachusetts General Hospital, UNITED STATES

## Abstract

**Background:**

Sustained retention in HIV care (RIC) and viral suppression (VS) are central to US national HIV prevention strategies, but have not been comprehensively assessed in criminal justice (CJ) populations with known health disparities. The purpose of this study is to identify predictors of RIC and VS following release from prison or jail.

**Methods and findings:**

This is a retrospective cohort study of all adult people living with HIV (PLWH) incarcerated in Connecticut, US, during the period January 1, 2007, to December 31, 2011, and observed through December 31, 2014 (*n =* 1,094). Most cohort participants were unmarried (83.7%) men (77.0%) who were black or Hispanic (78.1%) and acquired HIV from injection drug use (72.6%). Prison-based pharmacy and custody databases were linked with community HIV surveillance monitoring and case management databases. Post-release RIC declined steadily over 3 years of follow-up (67.2% retained for year 1, 51.3% retained for years 1–2, and 42.5% retained for years 1–3). Compared with individuals who were not re-incarcerated, individuals who were re-incarcerated were more likely to meet RIC criteria (48% versus 34%; *p <* 0.001) but less likely to have VS (72% versus 81%; *p =* 0.048). Using multivariable logistic regression models (individual-level analysis for 1,001 individuals after excluding 93 deaths), both sustained RIC and VS at 3 years post-release were independently associated with older age (RIC: adjusted odds ratio [AOR] = 1.61, 95% CI = 1.22–2.12; VS: AOR = 1.37, 95% CI = 1.06–1.78), having health insurance (RIC: AOR = 2.15, 95% CI = 1.60–2.89; VS: AOR = 2.01, 95% CI = 1.53–2.64), and receiving an increased number of transitional case management visits. The same factors were significant when we assessed RIC and VS outcomes in each 6-month period using generalized estimating equations (for 1,094 individuals contributing 6,227 6-month periods prior to death or censoring). Additionally, receipt of antiretroviral therapy during incarceration (RIC: AOR = 1.33, 95% CI 1.07–1.65; VS: AOR = 1.91, 95% CI = 1.56–2.34), early linkage to care post-release (RIC: AOR = 2.64, 95% CI = 2.03–3.43; VS: AOR = 1.79; 95% CI = 1.45–2.21), and absolute time and proportion of follow-up time spent re-incarcerated were highly correlated with better treatment outcomes. Limited data were available on changes over time in injection drug use or other substance use disorders, psychiatric disorders, or housing status.

**Conclusions:**

In a large cohort of CJ-involved PLWH with a 3-year post-release evaluation, RIC diminished significantly over time, but was associated with HIV care during incarceration, health insurance, case management services, and early linkage to care post-release. While re-incarceration and conditional release provide opportunities to engage in care, reducing recidivism and supporting community-based RIC efforts are key to improving longitudinal treatment outcomes among CJ-involved PLWH.

## Abstract 2

In a cohort study from the United States, Jaimie P. Meyer and colleagues identify predictors of retention in HIV care and viral suppression following release from prison or jail.

## Author summary

**Why was this study done?:**

- HIV prevention and treatment strategies aim to reduce HIV-related morbidity, mortality, and transmission by retaining people with HIV in care and sustaining them on antiretroviral treatment to achieve viral suppression (VS).
- Few prior studies had described long-term retention in HIV care (RIC) or VS for people incarcerated in prisons or jails and transitioning to communities. In fact, incarceration periods are often excluded from studies of RIC. This is an important knowledge gap because HIV and incarceration are overlapping epidemics that disproportionately affect people who are already marginalized by homelessness, substance use and psychiatric disorders, and socioeconomic status.

**What did the researchers do and find?:**

- We merged statewide databases from the Connecticut Department of Correction and Connecticut Department of Public Health on all people living with HIV who were released from prisons or jails in Connecticut, US, between 2007 and 2011. We followed each individual in this cohort for 3 years after release from prison/jail to examine RIC and VS.
- Among 1,094 individuals included in the study, continuous RIC declined over time (67.2% retained during year 1, 51.3% retained during years 1–2, and 42.5% retained during all 3 years). Compared with individuals who were not re-incarcerated, individuals who were re-incarcerated were more likely to meet RIC criteria (48% versus 34%; *p <* 0.001) but less likely to have VS (72% versus 81%; *p =* 0.048).
- Successful RIC and achievement of VS was associated with older age (RIC: adjusted odds ratio [AOR] = 1.61; VS: AOR = 1.37), having health insurance (RIC: AOR = 2.15; VS: AOR = 2.01), being treated for HIV while in prison (RIC: AOR = 1.33; VS: AOR = 1.91), receiving case management services during follow-up (RIC: AOR = 1.79; VS: AOR = 1.31), and early linkage to care in the community following release (RIC: AOR = 2.64; VS: AOR = 1.79). In addition, receiving an increased number of case management visits after release and spending an increased proportion of follow-up time re-incarcerated were correlated with better RIC and VS outcomes.

**What do these findings mean?:**

- Dedicated resources are needed to optimize people’s HIV care while they are in prison and to link them to care following release. Although prior studies suggest that prison provides a temporary window of opportunity to reconnect people to care, sustained retention in care and continuity of care ultimately require keeping people in the community longer and avoiding incarceration.

## Introduction

Along the HIV care continuum, retention in HIV care (RIC) is necessary for providing antiretroviral therapy (ART) and achieving viral suppression (VS), which reduces individual morbidity, mortality, and forward transmission [1–4]. Most incident HIV infections in the US are acquired from people living with HIV (PLWH) who are either undiagnosed or diagnosed but not retained in HIV care [5–7]. Poor RIC is associated with minority race/ethnicity, younger age, substance use disorders, and incarceration [8–12], although few studies have assessed longitudinal RIC beyond 6- or 12-month follow-up periods [13–16].

The US has the highest incarceration rate globally (910 per 100,000 adults) [17,18], with one-sixth of the country’s 1.2 million PLWH cycling through prisons or jails annually [19]. Yet incarcerated PLWH are frequently censored or excluded altogether from RIC studies [20]. For PLWH engaged in community-based care, frequent brief incarcerations disrupt care, and undermine ART adherence and VS [21–25]. When healthcare is optimized during incarceration, the highly structured environment can be an opportunity to reengage PLWH in care, initiate ART, and achieve VS, though this is often unsustained after release [26–28].

While several recent studies have elucidated challenges with linkage to community care post-release [29–32], the longitudinal impact of incarceration on continuity of HIV care remains poorly understood. Prior studies of RIC that have included criminal justice (CJ)–involved PLWH have been limited by short follow-up [33–35], exclusion of PLWH re-incarcerated during follow-up [34], recall biases in self-reported incarceration and ART use [9,21,24], reliance on ART prescription refill data [25], and inability to comprehensively link community and CJ data [26,28]. Because RIC is currently defined as having a clinic visit with viral load (VL) assessment at least every 6 months, a window of observation beyond 1 year is needed to better understand RIC [36]. Furthermore, a more nuanced understanding of longitudinal RIC among incarcerated PLWH is important for the development of future policies and interventions to address deficiencies within both CJ and community systems of care.

We therefore assessed 3-year RIC and VS in a large retrospective cohort of incarcerated PLWH. We had hypothesized that having health insurance and successfully linking to care would predict sustained RIC, but did not presuppose how recidivism would impact outcomes. Because we linked all community and CJ data within an integrated CJ system, we were able to examine “real world” outcomes in CJ-involved PLWH, accounting for re-incarcerations during follow-up and HIV-1 RNA levels obtained in both CJ and community settings.

## Methods

### Setting

The Connecticut Department of Correction (CTDOC) has been described previously [32]. Healthcare within CTDOC is guided by federally monitored clinical protocols requiring VL assessment within 96 hours of arrival, with continued monitoring every 3 months during incarceration. ART is prescribed according to national guidelines, which, at the time of observation, used CD4-based criteria.

### Data sources

As previously published [32], we combined comprehensive custody and pharmacy data from the CTDOC with the Connecticut Department of Public Health (CTDPH) Enhanced HIV/AIDS Reporting System (eHARS) surveillance and CAREWare service utilization databases. The eHARS surveillance system is maintained by CTDPH to be >95% complete. In the original data analysis plan, we prespecified linkage to and retention in care as major outcomes of interest (S1 Text).

### Study population

**Fig 1:**
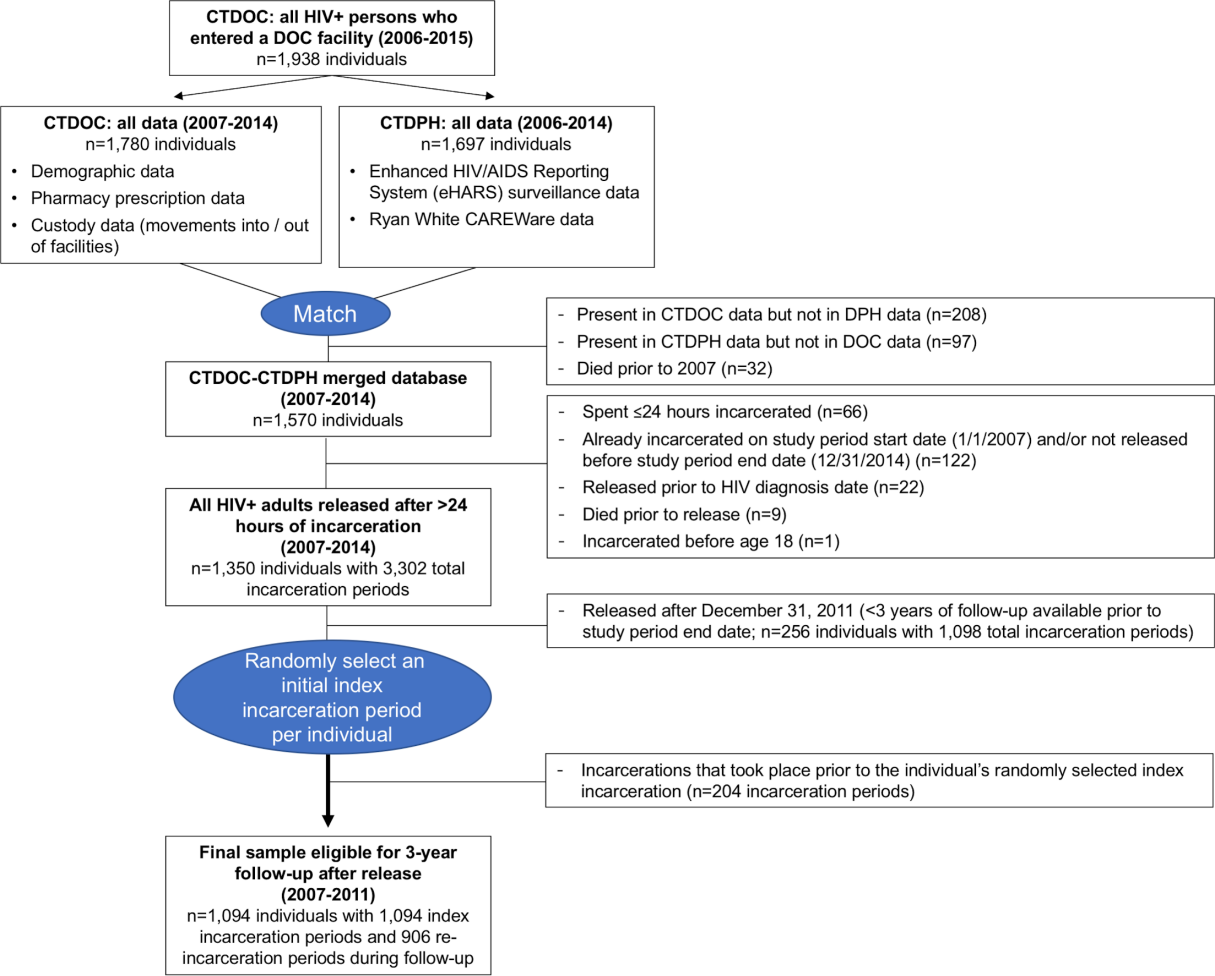
Participant flow diagram. CTDOC, Connecticut Department of Correction; CTDPH, Connecticut Department of Public Health; DOC, Department of Correction; DPH, Department of Public Health.

There were 1,094 individuals who met the following inclusion criteria (Fig 1): (1) were ≥18 years old with confirmed HIV before release from CTDOC; (2) were included in CTDOC and CTDPH databases; (3) were incarcerated at least once for >24 hours between January 1, 2007, and December 31, 2011; and (4) had ≥3 years of observation data after release (through December 31, 2014). For individuals never re-incarcerated, their only incarceration period was analyzed as their index incarceration. For participants with multiple eligible incarcerations (*n =* 538), we randomly selected 1 incarceration period to treat as each individual’s index incarceration/release to avoid differentially biasing the sample toward earlier incarceration periods (when fewer resources were available for HIV treatment and care) or later incarceration periods (with less time to observe outcomes or re-incarcerations). Random selection of index incarceration periods is consistent with an approach justified in prior studies of hospital readmissions and avoids inflating the association between re-incarceration during follow-up and the RIC and VS outcomes [37,38]. Subsequent re-incarcerations were recorded as covariates. Individuals entered the cohort starting on the day of index release from a CTDOC facility and were followed for 3 years or until death. For logistic regression models assessing outcomes after 3 years of follow-up, 1,001 PLWH were included, after excluding 93 deaths. For models using generalized estimating equations (GEEs), the full cohort of 1,094 PLWH contributed 6,227 complete 6-month follow-up periods (prior to death).

### Data merging

CTDOC databases were securely transferred to the CTDPH, where on-site data managers matched individuals to eHARS and CAREWare databases [32]. CTDOC inmate numbers are routinely reported to the CTDPH by facility nursing supervisors when a new HIV case is diagnosed in prison or by eHARS managers as part of routine data management. Inmate numbers were thus available for a majority of individuals in CTDPH databases. Rather than solely relying on inmate number, the match was done using the Link Plus probabilistic record linkage program developed by the Centers for Disease Control and Prevention (https://www.cdc.gov/cancer/npcr/tools/registryplus/lp.htm), with confirmatory data points including name, date of birth, race, and sex. The merged dataset was further restricted to PLWH currently living in Connecticut (excluding 97 individuals), then de-identified and securely provided to investigators for analysis (Fig 1).

### Measures

Recorded HIV-1 RNA VLs served as the proxy for routine HIV care clinic visits (both in prison/jail and in the community), which has demonstrated validity in multiple other settings and is consistent with core indicators for national HIV surveillance [39,40]. Using national RIC guidelines, we defined “sustained 3-year RIC” as having ≥1 VL measured during every 6-month period during the 3-year follow-up period, with ≥60 days between VLs in adjacent periods [36]. Because RIC does not necessarily predict VS and because VL was measured at different frequencies during follow-up, we created a “terminal VS” outcome, defined as having VL < 400 copies/ml measured within the last 6-month period of the 3-year follow-up [1,26]. Using time period as the unit of analysis, additional major outcomes were (1) “RIC over time” (defined as having ≥1 VL measured during a 6-month period) and (2) “VS over time” (defined as having ≥1 VL measured during a 6-month period with the last measured VL being <400 copies/ml). We concluded that the use of a binary outcome in accordance with the standard definitions of RIC would be more appropriate than a survival analysis strategy (which examines time to an event) and would thus provide more clinically meaningful results. PLWH without VL assessments at least every 6 months were defined as being out of care; missing VLs were conservatively assigned VL ≥ 400 copies/ml (non-suppression) by convention [41], which applied to 24.5% (245/1,001) of the individuals and 23.0% (1,432/6,227) of the analyzed 6-month periods in the final multivariable models [42]. VLs were not missing at random, and therefore multiple imputation was not performed. For example, compared to the 756 people with VLs recorded during the last 6-month period of observation, the 245 people with missing VLs were significantly more likely to be uninsured, to have been recently diagnosed with HIV, to have been diagnosed with HIV during index incarceration, to have had fewer re-incarcerations or less time spent re-incarcerated, and to have had fewer case management visits. There were no significant differences between individuals missing and not missing VLs in terms of age, race/ethnicity, or sex. Using the Behavioral Model for Vulnerable Populations framework [43], adapted for CJ populations [44], we examined a broad range of predisposing, enabling/disabling, and need severity factors as potential predictors of RIC and VS over time. Continuous variables that were not normally distributed were categorized or calculated as described below.

#### Predisposing factors

Predisposing factors included demographic characteristics (sex, race/ethnicity, education level, and marital status), source of HIV transmission, and time since HIV diagnosis. Sex was dichotomized as male/female based on available data; there was no consistent information available on the number of individuals who were transgender, intersex, or gender-nonconforming. Age was dichotomized at the sample median of 45 years. CTDPH databases assessed prior injection drug use (IDU) based on the original HIV risk, and time since HIV diagnosis was calculated by subtracting release date from HIV diagnosis date.

#### Enabling/Disabling factors

Enabling/disabling factors included year of release, whether HIV was diagnosed during the index incarceration, and health insurance coverage (dichotomous; time-varying in GEE models), which was assessed every 6–12 months in the CAREWare database and dichotomized as yes (public or private insurance) or no (“none”, “unknown”, or “not reported”); if healthcare or social service resources were used without having formal health insurance, persons were designated as uninsured. Using previous criteria, early linkage to care was defined as VL assessment within 14 days after index release [32]. Length of incarceration was calculated using dates and types of movements into and out of facilities and analyzed categorically. Generally, shorter incarcerations (≤30 days) corresponded to jail detentions, whereas longer incarcerations (≥365 days) involved prison sentences. Conditions of release were categorized as unsupervised, conditional release (e.g., parole or transitional housing), or release on bond. Because length of incarceration and conditions of release are closely associated, we created 1 multilevel categorical variable. Re-incarceration (recidivism) was defined as spending >24 hours in a CTDOC facility after initial release. To fully explore the potential effect of re-incarceration, we examined it in 4 ways: dichotomous (re-incarcerated during follow-up or not; time-varying in GEE models), categorical (number of times re-incarcerated during follow-up), total number of days spent in a CTDOC facility during the 3-year follow-up, and percentage of each 6-month period spent in a CTDOC facility (time-varying). Case management visit dates were used to create a dichotomous variable for receipt of case management services during each 6-month period (time-varying) and total number of case management visits over the 3-year follow-up period. CTDOC provides additional psychiatric case management services for those with serious mental illness, but these are not consistently recorded in CAREWare.

#### Need severity factors

The last VL measured during the index incarceration (within 90 days of release) was used to determine VS status prior to release. ART prescription during incarceration was extracted from pharmacy data and coded dichotomously. Prescribed medications to treat psychiatric disorders (i.e., antipsychotics, antidepressants, or other neuropsychiatric medications), treatment for an opioid use disorder (i.e., medication-assisted therapy with methadone, buprenorphine, or naltrexone as brief supervised withdrawal or maintenance therapy), and treatment of other medical comorbidities were each coded dichotomously. The number of medical conditions other than HIV treated during the incarceration period was summed [44]. As previously described [32], inmates are assigned psychiatric need and addiction severity scores on intake (5-point scale) to determine service programming, with 1–2 (no or low severity), 3 (moderate, requiring treatment), and 4–5 (severe, needing residential or intensive outpatient treatment). Increased psychiatric need was further assessed by combining psychiatric severity score and psychiatric disease treatment to create a 4-category psychiatric need variable (lower severity [score 1–2], untreated; lower severity, treated; higher severity [score 3–5], untreated; higher severity, treated) [32]. Additional information on psychiatric and substance use diagnoses was unavailable.

### Statistical analysis

To examine RIC and VS over time, Cochrane–Armitage tests for trend were used to compare the proportion of PLWH with RIC or with VS during year 1, years 1–2, and years 1–3. Chi-squared tests were used to compare RIC for re-incarcerated individuals and those who were not re-incarcerated. Among PLWH with RIC, we assessed the proportion with terminal VS using chi-squared tests. Logistic regression was used to model predictors of 3-year sustained RIC and terminal VS. Then, we examined each 6-month period of the 3-year follow-up period for RIC and VS over time using a logit GEE, assuming an autoregressive correlation structure to account for intra-individual correlation. Observations on the same individual were not assumed to be independent; rather, the GEE model allowed us to account for correlated release periods for the same individual and to calculate appropriate standard errors when performing statistical inference. Individuals who died during follow-up were excluded from logistic regression models but could contribute any complete 6-month time periods before death to the GEE models; including the incomplete periods during which PLWH died did not change effect estimates nor model fit.

For model building, relevant variables within the Behavioral Model for Vulnerable Populations with clinical significance or bivariate associations significant at *p <* 0.20 were included in full multivariable models. To minimize the Akaike information criterion and maximize the area under the receiver operating characteristic curve, backward selection was used to generate final parsimonious models, including variables with *p <* 0.05. Sex, race/ethnicity, and recidivism were assessed for significance in parsimonious models a priori. Final logistic regression models were also assessed for fit using Hosmer–Lemeshow goodness-of-fit tests (*p* > 0.05). Based on tolerance, variance inflation factor, and eigenvalue diagnostics, final models did not have significant multicollinearity. Interactions between race/ethnicity, sex, and recidivism were not found to be statistically significant. Due to small numbers of individuals treated for opioid withdrawal, this variable was only assessed in GEE models. All analyses were performed using SAS version 9.4 (SAS Institute).

### Ethics approval

The CTDOC Research Advisory Committee and institutional review boards at Yale University and CTDPH approved all procedures. Participant consent was waived because all data were previously collected and de-identified for analysis.

## Results

### Sample description

Table 1 summarizes selected characteristics of the included 1,094 PLWH. Half (52.3%) were >45 years old, and most were male (77.0%) and of racial/ethnic minorities (81.8%). Most HIV infections were related to IDU (72.6%) and were not recently diagnosed (96.1%).

**Table 1:** Description of the full sample of 1,094 individuals initially released from prison or jail during 2007–2011.

Variable	Full sample *n* (%)* (*n =* 1,094 individuals)
***Predisposing factors***	
**Age at time of release**	
≤45 years	422 (47.7%)
>45 years	572 (52.3%)
**Sex**^†^	
Female	252 (23.0%)
Male	842 (77.0%)
**Race/ethnicity**	
White	198 (18.1%)
Black	452 (41.2%)
Hispanic	404 (36.9%)
Other	41 (3.8%)
**Education level**	
<High school	508 (46.4%)
≥High school	586 (53.6%)
**Marital status**^‡^	
Not married	887 (83.7%)
Married	173 (16.3%)
**Injection-drug-use-related source of HIV transmission**	
No	300 (27.4%)
Yes	794 (72.6%)
**Time since HIV diagnosis**	
≤1 year	43 (3.9%)
>1 year	1,051 (96.1%)
***Enabling or disabling factors***	
**Any health insurance**	
No insurance/none reported	478 (43.7%)
Yes	616 (56.3%)
**HIV diagnosed during index incarceration**	
No	1,072 (98.0%)
Yes	22 (2.0%)
**Year of release**	
2007–2008	430 (39.3%)
2009–2010	469 (42.8%)
2011	195 (17.8%)
**Length of incarceration and conditions of release**	
Incarcerated ≤30 days, release without conditions	199 (18.2%)
Incarcerated ≤30 days, conditional or bonded release	144 (13.2%)
Incarcerated 31–364 days, release without conditions	383 (35.0%)
Incarcerated 31–364 days, conditional or bonded release	206 (18.8%)
Incarcerated ≥365 days, release without conditions	71 (6.5%)
Incarcerated ≥365 days, conditional release (none were released on bond)	91 (8.3%)
**Number of re-incarcerations**	
0	556 (50.8%)
1	274 (25.1%)
2	153 (14.0%)
≥3	111 (10.2%)
**Days spent re-incarcerated**	
0–6 (<1 week)	567 (51.8%)
7–30	52 (4.8%)
31–90	96 (8.8%)
91–180	171 (15.6%)
181–365	162 (14.8%)
>365	46 (4.2%)
**Number of transitional case management visits**	
0	599 (54.8%)
1–5	116 (10.6%)
6–14	162 (14.8%)
15–30	115 (10.5%)
>30	102 (9.3%)
**Early linkage to care (within 14 days of index release)**	
No	836 (76.4%)
Yes	230 (21.0%)
Re-incarcerated within 14 days	28 (2.6%)
***Need severity factors***	
**Prescribed ART during incarceration**	
No	458 (41.9%)
Yes	636 (58.1%)
**Virally suppressed prior to release**^**§**^	
No	487 (44.5%)
Yes	357 (32.6%)
Viral load not drawn prior to release	250 (22.9%)
**Number of medical comorbidities**^**||**^	
0	677 (61.9%)
1	232 (21.2%)
≥2	185 (16.9%)
**Psychiatric need**	
Lower severity score, untreated	505 (46.2%)
Lower severity score, treated	53 (4.8%)
Higher severity score, untreated	205 (18.7%)
Higher severity score, treated	331 (30.3%)
**Addiction severity score**^**¶**^	
1–2	163 (15.2%)
3	708 (66.0%)
4–5	201 (18.8%)
**Treated for an opioid use disorder during index incarceration**	
No	1,091 (99.7%)
Yes	3 (0.3%)

*Numbers listed are *n* (%) out of the total number of individuals who were initially eligible for analysis (*n =* 1,094), including those who were found to have died during follow-up (*n =* 93). Percentages may not sum to 100% due to rounding.

^†^Transgender males (*n =* 1) were included the male category, and transgender females (*n =* 2) were included in the female category.

^‡^There were *n =* 34 individuals with a missing or unreported marital status during their index incarceration.

^§^In 4% of cases, a viral load was drawn within 90 days prior to release, but the viral load value itself was not reported. These cases were included in the “no” viral suppression category because viral suppression could not be confirmed.

^||^Medical comorbidities broadly included gastrointestinal disease, cardiovascular disease, hyperlipidemia, diabetes, other endocrine disease, viral hepatitis C, hematologic disorders, hypercoagulable states, hypertension, immunological and autoimmune conditions, neurological conditions, pregnancy, pulmonary disease, renal failure, and urological conditions including benign prostatic hypertrophy.

^¶^There were *n =* 22 individuals whose addiction severity scores were never assessed during their index incarceration.

### Description of retention in care and viral suppression over 3 years

**Fig 2:**
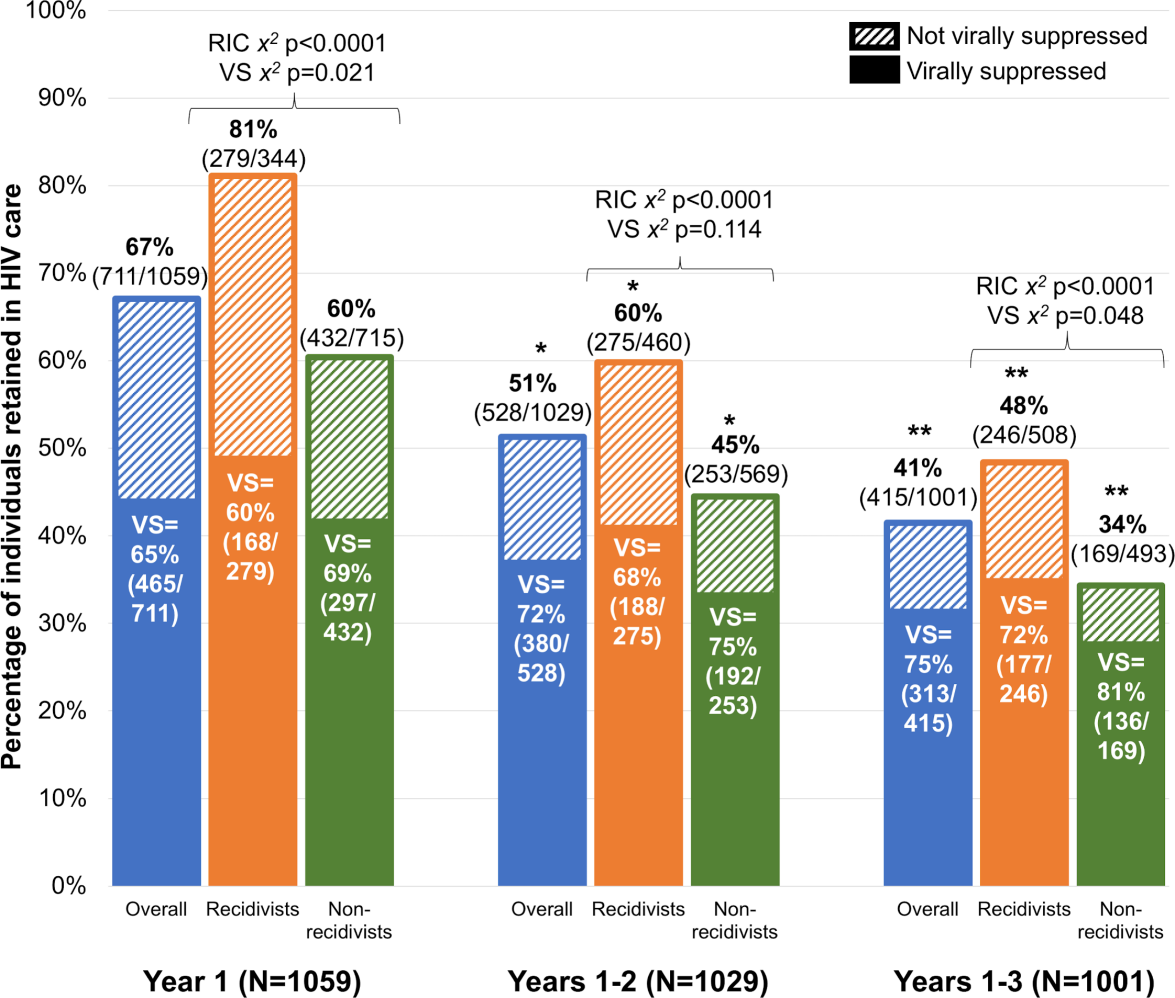
Longitudinal sustained retention in HIV care (RIC), based on frequency of HIV-1 RNA viral testing, and percentage with viral suppression (VS) at 1, 2, and 3 years post-release, stratified by whether individuals were re-incarcerated (recidivist) at some point during the follow-up period. There was a statistically significant difference in RIC rates between individuals were re-incarcerated and individuals who were not re-incarcerated across all time points (χ^2^ *p* < 0.001). Among those retained, individuals who were not re-incarcerated had higher VS rates compared to re-incarcerated individuals at end of year 1 (χ^*2*^ *p* = 0.021) and year 3 (χ^*2*^ *p* = 0.048). *Statistically significant decline in RIC compared with initial 1-year rates (McNemar’s test *p* < 0.001). **Statistically significant decline in RIC compared with sustained 2-year rates (McNemar’s test *p* < 0.001).

**Fig 3:**
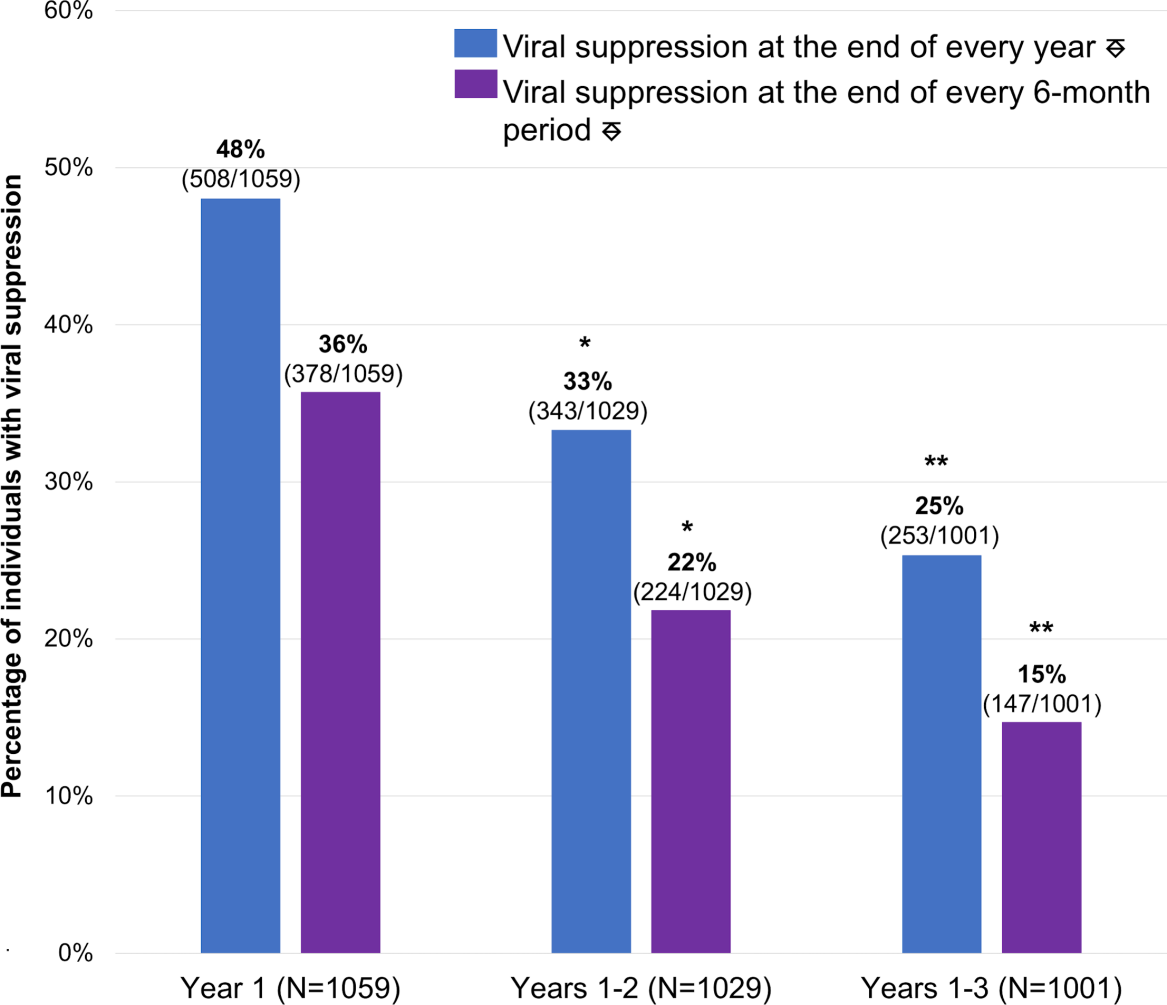
Longitudinal HIV viral suppression (VS) at 1, 2, and 3 years post-release. ^⎑^Individuals with detectable viral loads during these time frames were considered virally suppressed if their last viral load within the time frame of interest was <400 copies/ml. *Statistically significant decline in VS compared with initial 1-year rates (McNemar’s test *p* < 0.001). **Statistically significant decline in VS compared with sustained 2-year rates (McNemar’s test *p* < 0.001).

Continuous post-release RIC (i.e., having ≥1 VL measured during every 6-month period, with ≥60 days between VLs in adjacent periods) [36] and VS significantly declined with each additional year of follow-up (Figs 2 and 3, respectively). Excluding deaths (*n =* 35 in year 1, *n =* 30 in year 2, and *n =* 28 in year 3), RIC rates were significantly higher among individuals who were re-incarcerated compared with those who were not within each time frame (Fig 2). Among those retained, however, re-incarcerated individuals were less likely to be virally suppressed than individuals who were not re-incarcerated; this pattern was consistent across all 3 years but statistically significant in year 1 and years 1–3 only.

For all individuals, only re-incarcerated individuals, and only individuals who were not re-incarcerated, there was a statistically significant decline in RIC over time (Cochran–Armitage test 1-way *p <* 0.001). There was a statistically significant difference in RIC rate between individuals who were re-incarcerated and those who were not across all time points (χ^2^ *p <* 0.001). Among those retained, individuals who were not re-incarcerated had higher VS rates compared to re-incarcerated individuals at the end of year 1 (χ^2^ *p =* 0.021) and year 3 (χ^2^ *p =* 0.048).

Individuals with detectable viral levels during these time frames were considered virally suppressed if their last viral level within the time frame of interest was <400 copies/ml. For both definitions of VS (i.e., VS at the end of every year and at the end of every 6-month period), there was a statistically significant decline in sustained VS over time (Cochran–Armitage test 1-way *p <* 0.001).

### Factors predicting sustained retention in care and VS after 3 years

The 1,001 PLWH who were alive 3 years after release (*n =* 93 died) were demographically similar to the overall sample, and 41.5% of PLWH met criteria for sustained RIC (Table 2). In the final model, sustained RIC was independently associated with older age (>45 years), having health insurance, being re-incarcerated for >90 days during follow-up, receiving >30 case management visits, and being linked to care or re-incarcerated within 14 days after initial release. VS prior to release was not independently associated with RIC, although not having a VL measured prior to release was negatively associated with RIC.

**Table 2:** Logistic regression model of sustained retention in care and HIV viral suppression.*

Variable	Total *n* (%)^†^ (*n =* 1,001 individuals)	Sustained 3-year retention in care					Terminal viral suppression				
		Row *n* (%)^‡^ with retention	Unadjusted model OR (95% CI)	*p*-Value	Parsimonious adjusted model OR (95% CI)	*p*-Value	Row *n* (%)^§^ with viral suppression	Unadjusted model OR (95% CI)	*p*-Value	Parsimonious adjusted model OR (95% CI)	*p*-Value
***Predisposing factors***											
**Age at time of release**											
≤45 years	495 (49.5%)	179 (36.2%)	Referent		Referent		243 (49.1%)	Referent		Referent	
>45 years	506 (50.6%)	236 (46.6%)	1.54 (1.20–1.99)	<0.001	1.61 (1.22–2.12)	**<0.001**	302 (59.7%)	1.54 (1.20–1.97)	**<0.001**	1.37 (1.06–1.78)	**0.018**
**Sex**^**||**^											
Female	237 (23.7%)	87 (36.7%)	Referent				123 (51.9%)	Referent			
Male	764 (76.3%)	328 (42.9%)	1.30 (0.96–1.75)	0.090			422 (55.2%)	1.14 (0.85–1.53)	0.368		
**Race/ethnicity**											
White	177 (17.7%)	69 (39.0%)	Referent				102 (57.6%)	Referent			
Black	416 (41.6%)	175 (42.1%)	1.14 (0.79–1.63)	0.485			230 (55.3%)	0.91 (0.64–1.30)	0.600		
Hispanic	371 (36.1%)	154 (41.5%)	1.11 (0.77–1.60)	0.574			194 (52.3%)	0.81 (0.56–1.16)	0.242		
Other	37 (3.7%)	17 (46.0%)	1.33 (0.65–2.72)	0.433			19 (51.4%)	0.78 (0.38–1.58)	0.484		
**Education level**											
<High school	456 (45.6%)	187 (41.0%)	Referent				236 (51.8%)	Referent			
≥High school	545 (54.5%)	228 (41.8%)	1.04 (0.80–1.33)	0.792			309 (56.7%)	1.22 (0.95–1.57)	0.118		
**Marital status**^**¶**^											
Not married	814 (84.2%)	344 (42.3%)	Referent				442 (54.3%)	Referent			
Married	153 (15.8%)	62 (40.5%)	0.93 (0.66–1.32)	0.691			87 (56.9%)	1.11 (0.78–1.57)	0.561		
**Injection drug use**											
No	281 (28.1%)	107 (38.1%)	Referent				148 (52.7%)	Referent			
Yes	720 (71.9%)	308 (42.8%)	1.22 (0.92–1.61)	0.175			397 (55.1%)	1.11 (0.84–1.46)	0.480		
**Time since HIV diagnosis**											
≤1 year	42 (4.2%)	11 (26.2%)	Referent				16 (38.1%)	Referent			
>1 year	959 (95.8%)	404 (42.1%)	2.05 (1.02–4.13)	**0.044**			529 (55.2%)	2.00 (1.06–3.78)	**0.033**		
***Enabling or disabling factors***											
**Any health insurance**											
No insurance/none reported	419 (41.9%)	122 (29.1%)	Referent		Referent		175 (41.8%)	Referent		Referent	
Yes	582 (58.1%)	293 (50.3%)	2.47 (1.89–3.22)	**<0.001**	2.15 (1.60–2.89)	**<0.001**	370 (63.6%)	2.43 (1.88–3.15)	**<0.001**	2.01 (1.53–2.64)	**<0.001**
**HIV diagnosed during index incarceration**											
No	979 (97.8%)	410 (41.9%)	Referent				537 (54.9%)	Referent			
Yes	22 (2.2%)	5 (22.7%)	2.45 (0.90–6.69)	0.081			8 (36.4%)	2.13 (0.88–5.11)	0.092		
**Year of release**											
2007–2008	382 (38.2%)	136 (35.6%)	Referent				181 (47.4%)	Referent			
2009–2010	432 (43.2%)	185 (42.8%)	1.36 (1.02–1.80)	**0.036**			241 (55.8%)	1.40 (1.06–1.85)	**0.017**		
2011	187 (18.7%)	94 (50.3%)	1.83 (1.28–2.61)	**<0.001**			123 (65.8%)	2.13 (1.49–3.07)	**<0.001**		
**Length of incarceration and conditions of release**											
Incarcerated ≤30 days, release without conditions	175 (17.5%)	71 (40.6%)	Referent				88 (50.3%)	Referent			
Incarcerated ≤30 days, conditional or bonded release	125 (12.5%)	61 (48.8%)	1.40 (0.88–2.22)	0.157			74 (59.2%)	1.43 (0.90–2.28)	0.127		
Incarcerated 31–364 days, release without conditions	353 (35.3%)	138 (39.1%)	0.94 (0.65–1.36)	0.744			185 (52.4%)	1.09 (0.76–1.56)	0.646		
Incarcerated 31–364 days, conditional or bonded release	190 (19.0%)	76 (40.0%)	0.98 (0.64–1.48)	0.912			107 (56.3%)	1.28 (0.84–1.93)	0.249		
Incarcerated ≥365 days, release without conditions	70 (7.0%)	26 (37.1%)	0.87 (0.49–1.53)	0.620			41 (58.6%)	1.40 (0.80–2.45)	0.242		
Incarcerated ≥365 days, conditional release (none were released on bond)	88 (8.8%)	43 (48.9%)	1.40 (0.84–2.34)	0.201			50 (56.8%)	1.30 (0.78–2.18)	0.317		
**Number of re-incarcerations**											
0	493 (49.3%)	169 (34.3%)	Referent				251 (50.9%)	Referent			
1	250 (25.0%)	101 (40.4%)	1.30 (0.95–1.78)	0.102			142 (56.8%)	1.27 (0.93–1.72)	0.129		
2	147 (14.7%)	82 (55.8%)	2.42 (1.66–3.52)	**<0.001**			86 (58.5%)	1.36 (0.94–1.97)	0.106		
≥3	111 (11.1%)	63 (56.8%)	2.52 (1.66–3.83)	**<0.001**			66 (59.5%)	1.41 (0.93–2.15)	0.104		
**Days spent re-incarcerated**											
0–6 (<1 week)	502 (50.2%)	171 (34.1%)	Referent		Referent		254 (50.6%)	Referent			
7–30	46 (4.6%)	17 (37.0%)	1.14 (0.61–2.12)	0.693	1.29 (0.66–2.51)	0.456	26 (56.5%)	1.27 (0.69–2.33)	0.443		
31–90	89 (8.9%)	37 (41.6%)	1.38 (0.87–2.18)	0.173	1.47 (0.90–2.41)	0.122	49 (55.1%)	1.20 (0.76–1.88)	0.438		
91–180	163 (16.3%)	77 (47.3%)	1.73 (1.21–2.48)	**0.003**	1.92 (1.29–2.84)	**0.001**	95 (58.3%)	1.36 (0.95–1.95)	0.088		
181–365	155 (15.5%)	80 (51.6%)	2.07 (1.43–2.98)	**<0.001**	2.36 (1.51–3.66)	**<0.001**	89 (57.4%)	1.32 (0.92–1.89)	0.138		
>365	46 (4.6%)	33 (71.7%)	4.91 (2.52–9.58)	**<0.001**	5.82 (2.80–12.11)	**<0.001**	32 (69.6%)	2.23 (1.16–4.28)	**0.016**		
**Number of transitional case management visits**											
0	532 (53.2%)	183 (34.4%)	Referent		Referent		254 (47.7%)	Referent		Referent	
1–5	110 (11.0%)	40 (36.4%)	1.09 (0.71–1.67)	0.694	0.75 (0.47–1.19)	0.224	72 (65.5%)	2.07 (1.35–3.18)	**<0.001**	1.69 (1.09–2.63)	**0.020**
6–14	150 (15.0%)	73 (48.7%)	1.81 (1.25–2.61)	0.002	1.12 (0.74–1.68)	0.604	87 (58.0%)	1.51 (1.05–2.18)	**0.027**	1.23 (0.84–1.79)	0.295
15–30	111 (11.1%)	54 (48.7%)	1.81 (1.20–2.73)	0.005	0.83 (0.51–1.36)	0.462	61 (55.0%)	1.34 (0.89–2.01)	**0.168**	1.08 (0.70–1.65)	0.731
>30	98 (9.8%)	65 (66.3%)	3.76 (2.38–5.92)	<0.001	1.84 (1.11–3.03)	**0.017**	71 (72.5%)	2.88 (1.79–4.63)	**<0.001**	2.04 (1.25–3.34)	**0.005**
**Early linkage to care (within 14 days of index release)**											
No	774 (77.3%)	281 (36.3%)	Referent		Referent		408 (52.7%)	Referent			
Yes	205 (20.5%)	120 (58.5%)	2.48 (1.81–3.39)	**<0.001**	2.31 (1.65–3.24)	**<0.001**	125 (61.0%)	1.40 (1.02–1.92)	0.035		
Re-incarcerated within 14 days	22 (2.2%)	14 (63.6%)	3.07 (1.27–7.41)	**0.013**	2.63 (1.03–6.74)	**0.044**	12 (54.6%)	1.08 (0.46–2.52)	0.865		
***Need severity factors***											
**Prescribed ART during incarceration**											
No	415 (41.5%)	147 (35.4%)	Referent				191 (46.0%)	Referent		Referent	
Yes	586 (58.5%)	268 (45.7%)	1.54 (1.19–1.99)	**0.001**			354 (60.4%)	1.79 (1.39–2.31)	**<0.001**	1.39 (1.06–1.82)	**0.016**
**Virally suppressed prior to release**											
No	439 (43.9%)	186 (42.4%)	Referent		Referent		220 (50.1%)	Referent			
Yes	333 (33.3%)	150 (45.1%)	1.12 (0.84–1.49)	0.458	0.92 (0.67–1.26)	0.616	209 (62.8%)	1.68 (1.26–2.24)	**<0.001**		
Viral load not drawn prior to release	229 (22.9%)	79 (34.5%)	0.72 (0.51–1.00)	**0.049**	0.65 (0.45–0.93)	**0.020**	116 (50.7%)	1.02 (0.74–1.41)	0.894		
**Number of medical comorbidities**											
0	626 (62.5%)	237 (37.9%)	Referent				320 (51.1%)	Referent			
1	215 (21.5%)	97 (45.1%)	1.35 (0.99–1.85)	0.061			132 (61.4%)	1.52 (1.11–2.09)	**0.009**		
≥2	160 (16.0%)	81 (50.6%)	1.68 (1.19–2.39)	**0.004**			93 (58.1%)	1.33 (0.93–1.89)	0.114		
**Psychiatric need**											
Lower severity score, untreated	457 (45.7%)	181 (39.6%)	Referent				234 (51.2%)	Referent			
Lower severity score, treated	50 (5.0%)	21 (42.0%)	1.10 (0.61–2.00)	0.743			35 (70.0%)	2.22 (1.18–4.18)	0.371		
Higher severity score, untreated	187 (18.7%)	75 (40.1%)	1.02 (0.72–1.45)	0.906			103 (55.1%)	1.17 (0.83–1.64)	**0.013**		
Higher severity score, treated	307 (30.7%)	138 (45.0%)	1.25 (0.93–1.67)	0.142			173 (56.4%)	1.23 (0.92–1.65)	0.162		
Addiction severity score**											
1–2	158 (16.1%	52 (32.9%)	Referent				78 (49.4%)	Referent			
3	644 (66.7%	274 (42.6%)	1.51 (1.05–2.18)	**0.028**			359 (55.8%)	1.29 (0.91–1.83)	0.150		
4–5	179 (18.3%)	78 (43.6%)	1.57 (1.01–2.46)	**0.045**			96 (53.6%)	1.19 (0.77–1.82)	0.435		

*p*-Values in bold are statistically significant (< 0.05).

*Sample is restricted to individuals who were alive at the end of the 3-year follow-up period; there were 93 deaths, resulting in 1,001 individuals eligible for analysis, among whom 41.5% (415/1,001) were retained in care continuously for 3 years and 54.4% (545/1,001) had a viral load < 400 copies/ml at the end of the 3 years.

^†^Numbers listed are *n* (%) out of the total number of individuals (*n =* 1,001). Percentages may not sum to 100% due to rounding.

^‡^Numbers listed are the row *n* (%) of individuals who experienced the outcome of sustained retention in care. Percentages should not be expected to sum to 100%.

^§^Numbers listed are the row *n* (%) of individuals who experienced the outcome of viral suppression after 3 years of follow-up. Percentages should not be expected to sum to 100%.

^||^Transgender males (*n =* 1) were included the male category, and transgender females (*n =* 2) were included in the female category.

^¶^Incarceration periods for individuals with missing/unreported marital status (*n =* 34) were excluded from the bivariate analysis, such that the total *n =* 1,025.

**Incarceration periods where the addiction severity score was never assessed (*n =* 20) were excluded from the bivariate analysis, such that the total *n =* 1,039.

OR, odds ratio.

Overall, 54.4% of individuals demonstrated terminal VS after 3 years of follow-up (Table 2), which was independently associated with age > 45 years, having health insurance, and receiving increased numbers of case management visits. Unlike RIC, VS was not independently associated with the percentage of overall follow-up time spent re-incarcerated. In addition, although VS before release and early linkage to care were not significantly correlated with terminal VS, ART prescribed during incarceration was positively associated with terminal VS.

### Factors predicting retention in care and VS over time

The full cohort of 1,094 PLWH contributed 6,227 6-month follow-up periods, with 77.0% of periods meeting the criteria for RIC (Table 3). Independent correlates of RIC per 6-month period were age > 45 years, being diagnosed with HIV >1 year prior to release, having health insurance, having a short (≤30 days) initial incarceration period followed by conditional or bonded release, re-incarceration, increased proportion of follow-up time spent re-incarcerated, receipt of case management services, and early linkage to care post-release. Compared with having a short index incarceration with unconditional release (i.e., “time served”), being incarcerated for ≥1 year with unconditional release was associated with significantly poorer RIC. RIC was also significantly less likely during the final 6-month follow-up period after the index release. Regarding need severity factors, receiving ART and being treated for a medical comorbidity during incarceration were positively associated with RIC, while no VL obtained before release was negatively associated with RIC.

**Table 3:** Binomial generalized estimating equations of retention in care and viral suppression per 6-month follow-up period.*

Variable	Total *n* (%)^†^ (*n =* 6,227 6-month periods)	Retention in care over time					Viral suppression over time				
		Row *n* (%)^‡^ with retention	Unadjusted model OR (95% CI)	*p*-Value	Parsimonious adjusted model OR (95% CI)	*p*-Value	Row *n* (%)^§^ with viral suppression	Unadjusted model OR (95% CI)	*p*-Value	Parsimonious adjusted model OR (95% CI)	*p*-Value
***Predisposing factors***											
**Age at time of index release**											
≤45 years	3,020 (48.5%)	2,226 (73.7%)	Referent		Referent		1,331 (44.1%)	Referent		Referent	
>45 years	3,207 (51.5%)	2,569 (80.1%)	1.45 (1.20–1.76)	**<0.001**	1.30 (1.07–1.57)	**0.008**	1,836 (57.3%)	1.70 (1.44–2.00)	**<0.001**	1.44 (1.22–1.71)	**<0.001**
**Sex**^||^											
Female	1,454 (23.4%)	1,125 (77.4%)	Referent				693 (47.7%)	Referent			
Male	4,773 (76.7%)	3,670 (76.9%)	0.98 (0.79–1.21)	0.855			2,474 (51.8%)	1.17 (0.97–1.41)	0.102		
**Race/ethnicity**											
White	1,111 (17.8%)	849 (76.4%)	Referent				594 (53.5%)	Referent			
Black	2,584 (41.5%)	2,028 (78.5%)	1.13 (0.87–1.45)	0.369			1,315 (50.9%)	0.90 (0.71–1.13)	0.356		
Hispanic	2,299 (36.9%)	1,735 (75.5%)	0.94 (0.72–1.22)	0.617			1,127 (49.0%)	0.82 (0.65–1.04)	0.100		
Other	233 (3.7%)	183 (78.5%)	1.12 (0.65–1.92)	0.680			131 (56.2%)	1.11 (0.72–1.69)	0.644		
**Education level**											
<High school	2,850 (45.8%)	2,174 (76.3%)	Referent				1,433 (50.3%)	Referent			
≥High school	3,377 (54.2%)	2,621 (77.6%)	1.10 (0.91–1.33)	0.325			1,734 (51.4%)	1.07 (0.91–1.27)	0.411		
**Marital status**											
Not married	5,048 (83.8%)	3,906 (77.4%)	Referent				2,533 (50.2%)	Referent			
Married	975 (16.2%)	738 (75.7%)	0.89 (0.69–1.16)	0.401			516 (52.9%)	1.10 (0.87–1.39)	0.420		
**Injection drug use**											
No	1,733 (27.8%)	1,280 (73.9%)	Referent				751 (43.3%)	Referent		Referent	
Yes	4,494 (72.2%)	3,515 (78.2%)	1.27 (1.03–1.56)	**0.025**			2,416 (53.8%)	1.49 (1.23–1.81)	**<0.001**	1.31 (1.07–1.60)	**0.009**
**Time since HIV diagnosis**											
≤1 year	253 (4.1%)	155 (61.3%)	Referent		Referent		86 (34.0%)	Referent			
>1 year	5,974 (95.9%)	4,640 (77.7%)	2.22 (1.40–3.53)	**<0.001**	1.66 (1.05–2.62)	**0.029**	3,081 (51.6%)	2.13 (1.33–3.42)	**0.002**		
***Enabling or disabling factors***											
Health insurance**											
No insurance/none reported	4,267 (68.5%)	3,128 (73.3%)	Referent		Referent		1,966 (46.1%)	Referent		Referent	
Yes	1,960 (31.5%)	1,667 (85.1%)	1.60 (1.36–1.88)	**<0.001**	1.61 (1.34–1.94)	**<0.001**	1,201 (61.3%)	1.41 (1.25–1.60)	**<0.001**	1.18 (1.02–1.38)	**0.028**
**Length of index incarceration and conditions of index release**											
Incarcerated ≤30 days, release without conditions	1,106 (17.8%)	834 (75.4%)	Referent		Referent		519 (46.9%)	Referent		Referent	
Incarcerated ≤30 days, conditional release	78 (1.3%)	67 (85.9%)	2.22 (0.97–5.05)	0.058	2.29 (1.00–5.27)	**0.050**	52 (66.7%)	2.20 (0.96–5.02)	0.061	2.38 (1.08–5.28)	**0.033**
Incarcerated ≤30 days, bonded release	712 (11.4%)	573 (80.5%)	1.39 (0.96–2.02)	0.077	1.66 (1.14–2.40)	**0.008**	384 (53.9%)	1.33 (0.97–1.82)	0.072	1.58 (1.16–2.17)	**0.004**
Incarcerated 31–364 days, release without conditions	2,201 (35.4%)	1,652 (75.1%)	1.00 (0.75–1.32)	0.979	0.77 (0.58–1.02)	0.068	1,028 (46.7%)	1.03 (0.80–1.31)	0.840	0.76 (0.59–0.97)	**0.029**
Incarcerated 31–364 days, conditional release	1,062 (17.1%)	825 (77.7%)	1.19 (0.87–1.62)	0.282	0.80 (0.58–1.10)	0.169	572 (53.9%)	1.38 (1.04–1.82)	**0.025**	0.86 (0.65–1.14)	0.299
Incarcerated 31–364 days, bonded release	116 (1.9%)	92 (79.3%)	1.28 (0.64–2.58)	0.487	0.99 (0.52–1.89)	0.986	50 (43.1%)	0.85 (0.45–1.60)	0.620	0.67 (0.37–1.22)	0.193
Incarcerated ≥365 days, release without conditions	420 (6.7%)	312 (74.3%)	0.95 (0.62–1.44)	0.799	0.55 (0.36–0.84)	**0.006**	228 (54.3%)	1.38 (0.94–2.01)	0.099	0.69 (0.47–1.02)	0.060
Incarcerated ≥365 days, conditional release (none released on bond)	532 (8.5%)	440 (82.7%)	1.64 (1.09–2.46)	**0.018**	0.96 (0.62–1.48)	0.863	334 (62.8%)	2.03 (1.43–2.87)	**<0.001**	1.16 (0.82–1.63)	0.397
Re-incarcerated**											
No	5,325 (85.5%)	3,931 (73.8%)	Referent		Referent		2,696 (50.6%)	Referent		Referent	
Yes	902 (14.5%)	864 (95.8%)	5.24 (4.04–6.79)	**<0.001**	2.27 (1.44–3.58)	**<0.001**	471 (52.2%)	0.99 (0.86–1.13)	0.836	0.65 (0.51–0.81)	**<0.001**
Percent time spent re-incarcerated**											
0%	5,019 (80.6%)	3,653 (72.8%)	Referent		Referent		2,494 (49.7%)	Referent		Referent	
1%–50%	749 (12.0%)	698 (93.2%)	4.35 (3.31–5.71)	**<0.001**	2.56 (1.67–3.91)	**<0.001**	367 (49.0%)	0.98 (0.85–1.14)	0.812	1.38 (1.08–1.76)	**0.010**
51%–100%	459 (7.4%)	444 (96.7%)	8.69 (5.34–14.16)	**<0.001**	5.39 (3.15–9.22)	**<0.001**	306 (66.7%)	1.72 (1.40–2.11)	**<0.001**	2.52 (1.91–3.32)	**<0.001**
**Year of index release**											
2007–2008	2,405 (38.6%)	1,777 (73.9%)	Referent				1,049 (43.6%)	Referent		Referent	
2009–2010	2,677 (43.0%)	2,074 (77.5%)	1.24 (1.00–1.52)	**0.046**			1,418 (53.0%)	1.49 (1.24–1.80)	**<0.001**	1.04 (0.85–1.27)	0.712
2011	1,145 (18.4%)	944 (82.5%)	1.65 (1.25–2.16)	**<0.001**			700 (61.1%)	2.02 (1.60–2.56)	**<0.001**	1.60 (1.24–2.06)	**0.003**
Transitional case management services**											
No	5,126 (82.3%)	3,803 (74.2%)	Referent		Referent		2,489 (48.6%)	Referent		Referent	
Yes	1,101 (17.7%)	992 (90.1%)	2.32 (1.91–2.82)	**<0.001**	1.79 (1.44–2.22)	**<0.001**	678 (61.6%)	1.48 (1.28–1.70)	**<0.001**	1.31 (1.12–1.53)	**<0.001**
**Early linkage to care**											
No	4,798 (77.1%)	3,541 (73.8%)	Referent		Referent		2,277 (47.5%)	Referent		Referent	
Yes	1,296 (20.8%)	1,142 (88.1%)	2.77 (2.15–3.57)	**<0.001**	2.64 (2.03–3.43)	**<0.001**	824 (63.6%)	1.95 (1.59–2.39)	**<0.001**	1.79 (1.45–2.21)	**<0.001**
Re-incarcerated within 14 days without any community viral load	133 (2.1%)	112 (84.2%)	1.91 (0.82–4.47)	0.135	1.57 (0.68–3.63)	0.295	66 (49.6%)	1.07 (0.60–1.91)	0.811	1.05 (0.56–1.98)	0.874
**Time since index release**^††^											
0 to <6 months	1,080 (17.3%)	853 (79.0%)	Referent		Referent		522 (48.3%)	Referent		Referent	
6 to <12 months	1,059 (17.0%)	826 (78.0%)	0.95 (0.80–1.12)	0.519	1.02 (0.85–1.23)	0.802	511 (48.3%)	1.00 (0.89–1.12)	0.978	1.00 (0.87–1.15)	1.000
12 to <18 months	1,039 (16.7%)	796 (76.6%)	0.87 (0.73–1.04)	0.123	0.86 (0.71–1.05)	0.132	519 (50.0%)	1.06 (0.93–1.22)	0.357	1.04 (0.89–1.22)	0.607
18 to <24 months	1,029 (16.5%)	789 (76.7%)	0.87 (0.73–1.05)	0.139	0.88 (0.72–1.08)	0.228	535 (52.0%)	1.15 (1.00–1.33)	**0.058**	1.14 (0.96–1.35)	0.137
24 to <30 months	1,019 (16.4%)	775 (76.1%)	0.84 (0.70–1.01)	0.071	0.84 (0.68–1.04)	0.104	535 (52.5%)	1.18 (1.01–1.36)	**0.032**	1.14 (0.96–1.36)	0.133
30 to 36 months	1,001 (16.1%)	756 (75.5%)	0.83 (0.69–0.99)	**0.038**	0.81 (0.65–0.99)	**0.041**	545 (54.5%)	1.28 (1.10–1.48)	**0.001**	1.26 (1.06–1.49)	**0.010**
***Need severity factors***											
**Prescribed ART during index incarceration**											
No	2,577 (41.4%)	1,855 (72.0%)	Referent		Referent		994 (38.6%)	Referent		Referent	
Yes	3,650 (58.6%)	2,940 (80.6%)	1.63 (1.35–1.97)	**<0.001**	1.33 (1.07–1.65)	**0.011**	2,173 (59.5%)	2.46 (2.07–2.91)	**<0.001**	1.91 (1.56–2.34)	**<0.001**
**Virally suppressed prior to index releas**											
No	2,744 (44.1%)	2,112 (77.0%)	Referent		Referent		1,190 (43.4%)	Referent		Referent	
Yes	2,059 (33.1%)	1,653 (80.3%)	1.23 (0.98–1.54)	0.068	1.06 (0.84–1.33)	0.620	1,302 (63.2%)	2.37 (1.96–2.87)	**<0.001**	1.94 (1.59–2.37)	**<0.001**
Viral load not drawn prior to release	1,424 (22.9%)	1,030 (72.3%)	0.77 (0.61–0.97)	**0.029**	0.71 (0.56–0.91)	**0.006**	675 (47.4%)	1.19 (0.96–1.47)	0.117	1.18 (0.95–1.47)	0.130
**Number of medical comorbidities**											
0	3,874 (62.2%)	2,872 (74.1%)	Referent		Referent		1,818 (46.9%)	Referent			
1	1,334 (21.4%)	1,079 (80.9%)	1.49 (1.17–1.90)	**0.001**	1.29 (1.01–1.66)	**0.046**	750 (56.2%)	1.48 (1.21–1.83)	**<0.001**		
≥2	1,019 (16.4%)	844 (82.8%)	1.73 (1.33–2.24)	**<0.001**	1.29 (0.96–1.74)	0.096	599 (58.8%)	1.64 (1.31–2.07)	**<0.001**		
**Psychiatric need**											
Lower severity score, untreated	2,853 (45.8%)	2,146 (75.2%)	Referent				1,353 (47.4%)	Referent		Referent	
Lower severity score, treated	312 (5.0%)	255 (81.7%)	1.47 (0.97–2.23)	0.069			199 (63.8%)	2.06 (1.38–3.09)	**<0.001**	1.47 (0.97–2.21)	0.068
Higher severity score, untreated	1,166 (18.7%)	889 (76.2%)	1.04 (0.80–1.34)	0.772			598 (51.3%)	1.15 (0.91–1.45)	0.233	1.36 (1.07–1.72)	**0.011**
Higher severity score, treated	1,896 (30.5%)	1,505 (79.4%)	1.28 (1.02–1.60)	**0.035**			1,017 (53.6%)	1.31 (1.08–1.59)	**0.006**	1.07 (0.87–1.31)	0.510
**Addiction severity score during index incarceration**^‡‡^											
1–2	957 (15.7%)	685 (71.6%)	Referent				425 (44.4%)	Referent			
3	4,030 (66.0%)	3,129 (77.6%)	1.38 (1.07–1.79)	**0.013**			2,110 (52.4%)	1.38 (1.09–1.75)	**0.008**		
4–5	1,118 (18.3%)	874 (78.2%)	1.44 (1.05–1.97)	**0.024**			561 (50.2%)	1.29 (0.96–1.73)	0.091		
**Treated for an opioid use disorder during index incarceration**											
No	6,209 (99.7%)	4,779 (77.0%)	Referent				3,152 (50.8%)	Referent			
Yes	18 (0.3%)	16 (88.9%)	2.17 (0.35–13.42)	0.405			15 (83.3%)	4.84 (0.71–33.10)	0.108		

*p*-Values in bold are statistically significant (< 0.05).

*Sample is restricted to 6-month follow-up periods where individuals were alive at the end of the 6-month period. There were 6,227 6-month post-release periods (1,080 individual-based clusters) eligible for analysis, of which there were 4,795 (77.0%) 6-month post-release periods during which at least 1 viral load was drawn (retained in care) and 3,167 (50.9%) 6-month post-release periods in which the last viral level obtained was <400 copies/ml (virally suppressed).

^†^Numbers listed are *n* (%) out of the total number of 6-month time periods (*n =* 6,227). Percentages may not sum to 100% due to rounding.

^‡^Numbers listed are the row *n* (%) of 6-month time periods during which the individual experienced the outcome of retention in care. Percentages should not be expected to sum to 100%.

^§^Numbers listed are the row *n* (%) of 6-month time periods during which the individual experienced the outcome of viral suppression. Percentages should not be expected to sum to 100%.

^||^Transgender males (*n =* 1) were included the male category, and transgender females (*n =* 2) were included in the female category.

^¶^Follow-up periods for individuals with missing/unreported marital status (*n =* 204) were excluded from the bivariate analysis, such that the total *n =* 6,023.

**Variable refers to the 6-month interval rather than the individual or index incarceration.

^††^In a sensitivity analysis of probability of viral suppression over time (by Cochran–Armitage test), there was a significant trend toward higher probability of viral suppression with increased time since initial release.

^‡‡^Follow-up periods where the addiction severity score was never assessed (*n =* 122) were excluded from the bivariate analysis, such that the total *n =* 6,105.

OR, odds ratio.

VS was reported in 50.9% of the eligible 6-month periods (Table 3). In GEE models, independent correlates of VS per 6-month period were age > 45 years, IDU-related transmission risk, having health insurance, having a short index incarceration period (≤30 days) followed by conditional or bonded release, increased percentage of follow-up time spent re-incarcerated, receipt of case management services, and early linkage to care. Unlike for RIC, disabling factors for VS were re-incarceration and a medium length of incarceration (31–364 days) followed by unconditional release. VS was also significantly better for more contemporary releases and during the final 6-month follow-up period after the index release. Receipt of ART, VS, and untreated high psychiatric need during incarceration were need severity factors each positively associated with VS over time.

## Discussion

To our knowledge, this is one of the longest assessments of RIC and VS in a large cohort of individuals with HIV released from prison or jail. Despite HIV being a chronic condition that requires lifelong treatment, prior longitudinal RIC studies in the general population have not accounted for the complex impact of incarceration and the unique vulnerabilities it represents for many PLWH [8,11,14–16]. By comprehensively linking multiple CJ and community-based data sources, we were able to follow all CJ-involved PLWH statewide, including those re-incarcerated. We identified major correlates of optimal HIV treatment outcomes and found that the impact of re-incarceration is complex and dependent on time spent in facilities and conditions of release. These findings offer new insights into potential strategies to improve RIC and VS in CJ-involved PLWH.

Rates of sustained RIC and VS significantly declined over time, with re-incarcerated individuals demonstrating higher RIC rates than individuals who were not re-incarcerated, across all 3 years. Re-incarceration likely represents “forced” reengagement in care, but was not necessarily associated with VS itself. Rather, the length of time one spent in correctional facilities was associated with RIC and VS per 6-month interval and over the 3 years of observation. These findings speak not only to the potential for incarceration to facilitate reengagement in HIV care within a structured setting that can provide appropriate care and resources [21,25,26], but also to the potential for re-incarceration to interrupt HIV care. Re-incarceration itself was associated with worse VS outcomes, which is consistent with literature showing an association between incarceration, ART non-adherence, and virological failure [21,24,25]. Short-term benefits gained during incarceration appear to be outweighed by the long-term harm incarceration inflicts on physical and mental health, especially after release [45].

Individuals who were not re-incarcerated and who demonstrated RIC in the community had significantly higher VS rates compared with re-incarcerated individuals, underscoring the importance of better supporting community-based RIC through expanded enabling resources like case management and health insurance and minimizing recidivism, which is disruptive both medically and socially [46,47]. This finding is consistent with that from a recent study in North Carolina and Rhode Island showing that PLWH released from prison and retained in community care (without being re-incarcerated) had similar VS rates to PLWH continuously engaged in community care [48]. Sentencing policies, particularly for drug-related or nonviolent offenses, should be modified to encourage community-based CJ rehabilitation and engagement in community-based healthcare and to facilitate access to post-release resources like psychiatric and addiction treatment, both of which improve RIC and reduce recidivism [49–54].

Engaging PLWH in the HIV care continuum during and immediately after release significantly impacts longitudinal RIC. PLWH whose VLs were adequately monitored, who were prescribed ART, or who achieved VS before release had better RIC over time. Also, early linkage to care (within 2 weeks) post-release was associated with sustained 3-year RIC as well as RIC and VS over time. Paradoxically, prisons/jails influence longitudinal HIV treatment outcomes, especially when community-based resources are inadequate. Many PLWH likely benefit from CJ-based services as a safety net as long as these services are integrated, continuous, and align health and justice priorities. If jail/prison services are comprehensive and coordinated, jails and prisons can serve as highly effective “patient-centered medical homes” [55]. Despite these opportunities, the uneven and often disjointed care provided in CJ settings and the detrimental medical and social consequences alongside the excessive financial burden associated with mass incarceration in the US favor supporting less costly, integrated community healthcare systems to improve care for PLWH [45,54,56–59].

Having a short index incarceration with subsequent supervised release was associated with increased RIC and VS over time relative to both short and longer incarcerations with unconditional release. PLWH with brief incarcerations may not lose their social and medical community-based ties [60] and consequently, with post-release support from CJ supervision, may better reintegrate back into the community [58]. Conditional release may also facilitate RIC by providing an access point for PLWH to engage in social and medical services, whereas PLWH released on bond may represent a population with greater financial resources or social support that improves their ability to navigate the healthcare system [29].

In randomized trials, transitional case management services for incarcerated PLWH are no better than pre-release discharge planning at improving post-release linkage to care and retention [32,33,61]. Within an integrated prison/jail system, and when targeted to those most in need, case management may require a differentiated service delivery model that caters to PLWH at highest risk for recidivism. Differentiated service delivery is a client-centered approach that simplifies and targets key services (e.g., health insurance and treatment for addiction and psychiatric disorders) needed along the HIV continuum to reduce unnecessary burdens on the health system [33,34,62]. In the absence of such services, multiple stressors and barriers to care can lead to substance use relapse, high-risk behaviors, and suboptimal healthcare engagement, such as defaulting from ART, which undermine VS [63–65]. Unlike prior studies, findings here demonstrate that transitional case management is a key enabling factor that is strongly associated with RIC and VS. Despite the important role of case management to facilitate health insurance and community services to improve RIC and VS [32,65], most PLWH (54.8%) did not receive these services, and health insurance coverage remained low (56.3%) over 3 years of follow-up. This indicates an urgent need to expand the provision of case management services both during and after the transition to the community.

When RIC and VS did not significantly improve despite numerous case management visits, it is likely that those PLWH had multiple severe medical and social needs. Thus, the positive effect of case management may be masked by the higher baseline need of those who received these services compared with those who were not targeted to receive case management. Unlike in Connecticut, most states terminate insurance benefits during incarceration [62], with findings here supporting the need to reexamine policies that promote continuation of, reactivation of, or potentially new enrollment into insurance before release.

Unlike previous studies [20,56], IDU transmission risk and high psychiatric need correlated with VS over time. While IDU and psychiatric need were not associated with frequency of transitional case management utilization, such individuals may have received additional psychiatric case management to link and retain them in treatment for psychiatric or substance use disorders, which could have improved VS. Also, some PLWH with an IDU history died early during follow-up, including from drug overdose [66], which limited our ability to clearly assess the role of current or past IDU on longitudinal HIV treatment outcomes.

Other limitations of the study included limited data regarding post-release housing status and psychiatric and substance use disorders. Addiction and psychiatric severity scores were our best indicators for comorbidities that potentially impact RIC in the community. We also could not fully measure brief fluctuations in insurance status.

Strengths of the study included the ability to follow both individuals who were re-incarcerated and those who were not, for an extended period of time, and to account for many factors that changed over time, including health insurance status. Instead of using prescription refill or clinic visit data to approximate RIC and VS, our outcomes were constructed using reliably and systematically reported biological data and used standardized, generalizable, and clinically justifiable definitions of RIC and VS. Defining missing VL data as indicating being out of care and not having VS may have biased findings, but is a standardized analytic convention that provides conservative estimates [40,41,67], given that a very small proportion of PLWH may have moved out of state and not been fully measured despite extensive efforts by CTDPH to cross-check interstate databases. Finally, we minimized typical database linkage challenges through the use of complete databases (aside from psychiatric case management data), reliable variables for individual matching, and CTDPH database managers with considerable experience linking data.

Despite some limitations, this study is, to our knowledge, one of the first to extensively identify correlates of longitudinal RIC and VS for all PLWH in a CJ setting, while simultaneously describing and accounting for the complex impact of incarceration. RIC decreases markedly after release from prison/jail, but several key factors correlate with improved RIC and VS after release, including provision of HIV care during incarceration, health insurance, case management, and early linkage to care post-release. While re-incarceration and conditional release facilitate engagement in care for some PLWH, our findings strongly indicate that strategies that reduce recidivism and support community-based RIC will yield better treatment outcomes than using re-incarceration as a mechanism to promote RIC in this population. Improving RIC and VS will, however, require policy changes, including expanding health insurance through new enrollments and avoiding suspension; expanding and targeting transitional case management to those at risk for recidivism and poor health outcomes; aligning community supervision (i.e., probation and parole) with healthcare by promoting continued care for HIV, psychiatric disorders, and addiction (which often requires health insurance) to avoid recidivism; and screening for and treating psychiatric and substance use disorders, and continuing these treatments post-release. Such changes in policy will likely positively influence HIV treatment outcomes while diminishing the negative consequences of mass incarceration, especially for racial/ethnic minorities in the US.

## Supporting information

S1 Checklist STROBE checklist. (DOC)

S1 Table Characteristics of all 1,094 individuals and their incarceration experiences, stratified based on the frequency of transitional case management services provided during the 3-year follow-up. (DOCX)

S1 Text Signed applications for protocol approval from CTDOC and CTDPH, including data on planned analyses. (PDF)

## Abbreviations

AOR: adjusted odds ratio
ART: antiretroviral therapy
CJ: criminal justice
CTDOC: Connecticut Department of Correction
CTDPH: Connecticut Department of Public Health
eHARS: Enhanced HIV/AIDS Reporting System
GEE: generalized estimating equation
IDU: injection drug use
PLWH: people living with HIV
RIC: retention in HIV care
VL: viral load
VS: viral suppression
