# Supplementary material for: Retention in HIV care during the 3 years following release from incarceration: A cohort study
Source: PLoS Med. 2018 Oct 9;15(10):e1002667. doi: 10.1371/journal.pmed.1002667 (PMC6177126; doi:10.1371/journal.pmed.1002667)
Supplement: S1 Table — (DOCX) [file pmed.1002667.s002.docx]

**S1 Table. Characteristics of all 1,094 individuals and their incarceration experiences, stratified based on the frequency of transitional case management services provided during 3-year follow-up ***

|  | **0**  **visits/year** | **1-5**  **visits/year** | **6-14 visits/year** | **15-30 visits/year** | **>30 visits/year** |  |
| --- | --- | --- | --- | --- | --- | --- |
|  | **n=532 (53.2%)** | **n=110 (11.0%)** | **n=150 (15.0%)** | **n=111 (11.1%)** | **n=98 (9.8%)** | ***p*-value** |
| **Retained in care for 3 years after release** **†**  No  Yes | 349 (65.6%)  183 (34.4%) | 70 (63.6%)  40 (36.4%) | 77 (51.3%)  73 (48.7%) | 57 (51.4%)  54 (48.7%) | 33 (33.7%)  65 (66.3%) | **<0.001** |
| **Viral suppression 3 years after release** **†**  No  Yes | 278 (52.3%)  254 (47.7%) | 38 (34.6%)  72 (65.5%) | 63 (42.0%)  87 (58.0%) | 50 (45.1%)  61 (55.0%) | 27 (27.6%)  71 (72.5%) | **<0.001** |
| ***Predisposing factors*** | **n=599 (54.8%)** | **n=116 (10.6%)** | **n=162 (14.8%)** | **n=115 (10.5%)** | **n=102 (9.3%)** | ***p*-value** |
| **Age at time of release**  ≤ 45 years  > 45 years | 297 (49.6%)  302 (50.4%) | 50 (43.1%)  66 (56.9%) | 82 (50.6%)  80 (49.4%) | 54 (47.0%)  61 (53.0%) | 39 (38.2%)  63 (61.8%) | 0.194 |
| **Gender ‡**  Female  Male | 168 (28.1%)  431 (72.0%) | 16 (13.8%)  100 (86.2%) | 33 (20.4%)  129 (79.6%) | 19 (16.5%)  96 (83.5%) | 16 (15.7%)  86 (84.3%) | **<0.001** |
| **Race/Ethnicity**  White  Black  Hispanic  Other | 111 (18.5%)  245 (40.9%)  216 (36.1%)  27 (4.5%) | 24 (20.7%)  46 (39.7%)  42 (36.2%)  4 (3.5%) | 31 (19.1%)  66 (40.7%)  59 (36.4%)  6 (3.7%) | 19 (16.5%)  52 (45.2%)  43 (37.4%)  1 (0.9%) | 13 (12.8%)  42 (41.2%)  44 (43.1%)  3 (2.9%) | 0.81 |
| **Education level**  < High school  ≥ High school | 277 (46.2%)  322 (53.8%) | 45 (38.8%)  71 (61.2%) | 73 (45.1%)  89 (54.9%) | 52 (45.2%)  63 (54.8%) | 61 (59.8%)  41 (40.2%) | **0.036** |
| **Marital status §**  Not married  Married | 469 (81.3%)  108 (18.7%) | 94 (83.9%)  18 (16.1%) | 129 (81.7%)  29 (18.4%) | 104 (93.7%)  7 (6.3%) | 91 (89.2%)  11 (10.8%) | **0.010** |
| **Injection drug use-related source of HIV transmission**  No  Yes | 163 (27.2%)  436 (72.8%) | 29 (25.0%)  87 (75.0%) | 43 (26.5%)  119 (73.5%) | 46 (40.0%)  69 (60.0%) | 19 (18.6%)  83 (81.4%) | **0.009** |
| **Time since HIV diagnosis**  ≤ 1 year  > 1 year | 25 (4.2%)  574 (95.8%) | 2 (1.7%)  114 (98.3%) | 10 (6.2%)  152 (93.8%) | 2 (1.7%)  113 (98.3%) | 4 (3.9%)  98 (96.1%) | 0.30 |
| ***Enabling or disabling factors*** |  |  |  |  |  |  |
| **Any health insurance**  No insurance/none reported  Yes | 338 (56.4%)  261 (43.6%) | 42 (36.2%)  74 (63.8%) | 48 (29.6%)  114 (70.4%) | 33 (28.7%)  82 (71.3%) | 17 (16.7%)  85 (83.3%) | **<0.001** |
| **HIV diagnosed during index incarceration**  No  Yes | 589 (98.3%)  10 (1.7%) | 114 (98.3%)  2 (1.7%) | 158 (97.5%)  4 (2.5%) | 113 (98.3%)  2 (1.7%) | 98 (96.1%)  4 (3.9%) | 0.60 |
| **Year of release**  2007-2008  2009-2010  2011 | 264 (44.1%)  250 (41.7%)  85 (14.2%) | 52 (44.8%)  38 (32.8%)  26 (22.4%) | 55 (34.0%)  76 (46.9%)  31 (19.1%) | 37 (32.2%)  49 (42.6%)  29 (25.2%) | 22 (21.6%)  56 (54.9%)  24 (23.5%) | **<0.001** |
| **Length of incarceration and conditions of release**  Incarcerated ≤ 30 days,  release without conditions  Incarcerated ≤ 30 days,  conditional or bonded release  Incarcerated 31-364 days,  release without conditions  Incarcerated 31-364 days,  conditional or bonded release  Incarcerated ≥ 365 days,  release without conditions  Incarcerated ≥ 365 days,  conditional release (none  were released on bond) | 388 (64.8%)  35 (5.8%)  60 (10.0%)  75 (12.5%)  32 (5.3%)  9 (1.5%) | 70 (60.3%)  4 (3.5%)  8 (6.9%)  16 (13.8%)  14 (12.1%)  4 (3.5%) | 54 (33.3%)  7 (4.3%)  15 (9.3%)  38 (23.5%)  42 (25.9%)  6 (3.7%) | 14 (12.2%)  4 (3.5%)  7 (6.1%)  25 (21.7%)  54 (47.0%)  11 (9.6%) | 41 (40.2%)  2 (2.0%)  6 (5.9%)  17 (16.7%)  20 (19.6%)  16 (15.7%) | **<0.001** |
| **Number of re-incarcerations**  0  1  2  ≥ 3 | 379 (63.3%)  146 (24.4%)  50 (8.4%)  24 (2.0%) | 70 (60.3%)  20 (17.2%)  13 (11.2%)  13 (11.2%) | 52 (32.1%)  54 (33.3%)  36 (22.2%)  20 (12.4%) | 14 (12.2%)  38 (33.0%)  38 (33.0%)  25 (21.7%) | 41 (40.2%)  16 (15.7%)  16 (15.7%)  29 (28.4%) | **<0.001** |
| **Days spent re-incarcerated**  0-6 (< 1 week)  7-30  31-90  91-180  181-365  > 365 | 388 (64.8%)  35 (5.8%)  60 (10.0%)  75 (12.5%)  32 (5.3%)  9 (1.5%) | 70 (60.3%)  4 (3.5%)  8 (6.9%)  16 (13.8%)  14 (12.1%)  4 (3.5%) | 54 (33.3%)  7 (4.3%)  15 (9.3%)  38 (23.5%)  42 (25.9%)  6 (3.7%) | 14 (12.2%)  4 (3.5%)  7 (6.1%)  25 (21.7%)  54 (47.0%)  11 (9.6%) | 41 (40.2%)  2 (2.0%)  6 (5.9%)  17 (16.7%)  20 (19.6%)  16 (15.7%) | **<0.001** |
| **Linked to care within 14 days**  No  Yes  Re-incarcerated prior to 14-  day linkage | 480 (80.1%)  108 (18.0%)  11 (1.8%) | 82 (70.7%)  31 (26.7%)  3 (2.6%) | 124 (76.5%)  30 (18.5%)  8 (4.9%) | 85 (73.9%)  26 (22.6%)  4 (3.5) | 65 (63.7%)  35 (34.3%)  2 (2.0%) | **0.004** |
| ***Need factors*** |  |  |  |  |  |  |
| **Prescribed ART during incarceration**  No  Yes | 302 (50.4%)  297 (49.6%) | 33 (28.5%)  83 (71.6%) | 53 (32.7%)  109 (67.3%) | 44 (38.3%)  71 (61.7%) | 26 (25.5%)  76 (74.5%) | **<0.001** |
| **Virally suppressed prior to release \|\|**  No  Yes  Viral load not drawn prior to  release | 273 (45.6%)  170 (28.4%)  156 (26.0%) | 51 (44.0%)  42 (36.2%)  23 (19.8%) | 62 (38.3%)  70 (43.2%)  30 (18.5%) | 58 (50.4%)  36 (31.3%)  21 (18.3%) | 43 (42.2%)  39 (38.2%)  20 (19.6%) | **0.015** |
| **Number of medical comorbidities ¶**  0  1  ≥ 2 | 401 (66.9%)  109 (18.2%)  89 (14.9%) | 65 (56.0%)  26 (22.4%)  25 (21.6%) | 92 (56.8%)  39 (24.1%)  31 (19.1%) | 64 (55.7%)  29 (25.2%)  22 (19.1%) | 55 (53.9%)  29 (28.4%)  18 (17.7%) | **0.042** |
| **Psychiatric need**  Low severity score,  untreated  Low severity score,  treated  High severity score,  untreated  High severity score,  treated | 288 (48.1%)  125 (20.9%)  19 (3.2%)  167 (27.9%) | 54 (46.6%)  13 (11.2%)  10 (8.6%)  39 (33.6%) | 71 (43.8%)  27 (16.7%)  11 (6.8%)  53 (32.7%) | 53 (46.1%)  19 (16.5%)  8 (7.0%)  35 (30.4%) | 39 (38.2%)  21 (20.6%)  5 (4.9%)  37 (36.3%) | 0.064 |
| **Addiction severity score ****  1-2  3  4-5 | 93 (16.0%)  380 (65.2%)  110 (18.9%) | 14 (12.1%)  79 (68.1%)  23 (19.8%) | 25 (15.7%)  102 (64.2%)  32 (20.1%) | 18 (15.9%)  76 (67.3%)  19 (16.8%) | 13 (12.9%)  71 (70.3%)  17 (16.8%) | 0.96 |
| **Treated for an opioid use disorder during index incarceration**  No  Yes | 598 (99.8%)  1 (0.2%) | 116 (100.0%)  0 (0.0%) | 162 (100%)  0 (0%) | 114 (99.1%)  1 (0.9%) | 101 (99.0%)  1 (1.0%) | 0.170 |

***** Total sample is the 1,094 individuals who were initially eligible for analysis (n=1094), including those who were found to have died during follow-up (n=93). Numbers listed are n (%) of the total sample or column n (%). *P*-values are generated from chi-squared tests or, for tables with at least one cell n<5, fisher’s exact tests generated using Monte Carlo estimations based on n=20,000 randomly chosen tables. Percentages may not sum to 100% due to rounding.

**†** Outcomes were assessed for the 1,001 individuals who were still alive after three years of follow-up.

**‡** Incarceration periods for transgender males (n=1) and transgender females (n=3) have been included in the male and female categories, respectively.

**§** There were n=34 individuals with a missing or unreported marital status during their index incarceration.

**||** In 4% of cases, a viral level was drawn within 90 days prior to release but the viral level value itself was not reported. These cases were included in the “No” viral suppression category because viral suppression could not be confirmed.

**¶** Medical comorbidities broadly included gastrointestinal disease, cardiovascular disease, hyperlipidemia, diabetes, other endocrine disease, viral hepatitis C, hematologic disorders, hypercoagulable states, hypertension, immunologic and autoimmune conditions, neurological conditions, pregnancy, pulmonary disease, renal failure, and urologic conditions including benign prostatic hypertrophy.

****** There were n=22 individuals whose addiction severity scores were never assessed during their index incarceration.
