# Supplementary material for: Retention in HIV care during the 3 years following release from incarceration: A cohort study
Source: PLoS Med. 2018 Oct 9;15(10):e1002667. doi: 10.1371/journal.pmed.1002667 (PMC6177126; doi:10.1371/journal.pmed.1002667)
Supplement: S1 Text — (PDF) [file pmed.1002667.s003.pdf]

PROPOSED RESEARCH TO BE REVIEWED BY THE
STATE OF CONNECTICUT DEPARTMENT OF PUBLIC HEALTH HUMAN INVESTIGATIONS COMMITTEE

DATE SUBMITTED: 12/01/2015

TITLE OF STUDY: The Impact of Incarceration and Release from a Correctional Facility on Linkage to and Retention in HIV Care and Viral Suppression among People Living With HIV in Connecticut
(Amended to Yale HIC proposal entitled: Effect of Newer Antiretroviral Regimens on HIV Biological Outcomes in HIV-infected Prisoners: A 13-Year Retrospective Evaluation)

PRINCIPAL INVESTIGATOR: Jaimie Meyer, MD, MS, Assistant Professor of Medicine (AIDS), Yale AIDS Program, Yale University School of Medicine; Assistant Clinical Professor of Nursing, Yale School of Nursing

CO-INVESTIGATOR: Frederick L Altice, MD, MA, Professor of Medicine (Infectious Diseases), Yale University School of Medicine; Professor of Epidemiology (Microbial Diseases), Yale University School of Public Health; Director, Clinical and Community Research; Director, HIV in Prisons Program

ADDRESS: Yale AIDS Program, 135 College St, Suite 323, New Haven, CT 06510

ORGANIZATION: Yale School of Medicine

OTHER INVESTIGATORS: Kelsey B Loeliger, MD-PhD Predoctoral Candidate; Epidemiology of Microbial Diseases Department, Yale University School of Public Health; Yale AIDS Program, Yale University School Medicine

Biographical Sketch for PI/Co-PI (PHS Form 398 may also be obtained on the internet at http://ocga2.ucsd.edu/nih/B.html) -**Please limit to 3 pages.** Please do not submit a curriculum vitae.

A signed HIC Confidentiality Pledge for individuals who will have access to identifiable health data.

☒ **This data request has been discussed with Heidi Jenkins (name of Department of Public Health (DPH) program employee) of the DPH program responsible for the data collection.**

☐ **This data request has not been discussed with a DPH Program employee.**

RESEARCH PROPOSAL OUTLINE

Sections:

Summary

One in seven people living with HIV (PLH) in the U.S. cycle throughout the criminal justice system each year. While studies show that up to 70% of inmates living with HIV who are prescribed ART can achieve viral suppression during their incarceration, this benefit is not sustained after release into the community. People interfacing with the criminal justice system experience comorbid medical, psychiatric, and social instabilities that put them at risk of care disengagement. In order to help decrease HIV-related morbidity and mortality among former inmates with HIV and to reduce

HIV transmission to their community contacts, more information is needed regarding longitudinal HIV outcomes as inmates cycle between the criminal justice system and the community.

Connecticut (CT) is unusual in that it has an integrated correctional health system which, combined with CT's statewide HIV/AIDS surveillance monitoring system (eHARS), provides data to examine interactions between HIV continuity of care and criminal justice involvement. By linking multiple CT Department of Correction databases with the CT DPH eHARS, this project aims to determine the relationship between incarceration, community release, and the continuity of HIV care. By using a retrospective cohort of PLH released from CT prisons and jails between 2007-2015, the broad aims for this application are to: **(1)** assess the time to linkage to HIV care and loss to clinic follow-up after release from prison or jail to the community; **(2)** evaluate the proportion of inmates achieving and maintaining viral suppression following release from prison or jail to the community, and identify time points of loss of suppression that could be potentially targeted by future interventions; and **(3)** compare longitudinal trends in HIV viral suppression during incarceration and time in the community, and identify groups of individuals demonstrating trends indicative of poor HIV outcomes. Exploratory aims are to identify and describe HIV outcomes for particularly high-risk sub-populations (i.e., women and ethnic/racial minorities).

The proposed study will identify key time points at which former inmates are at the highest risk for developing poor HIV outcomes (i.e., loss to follow-up and loss of viral suppression) and the times at which community- and criminal justice system-based healthcare interventions would be most beneficial. The innovative linkage of community-based health data and integrated Department of Correction custody and pharmacy data at a statewide level will allow for one of the first studies to follow both recidivists and non-recidivists in the community and to use longitudinal methods to evaluate HIV outcomes after release into the community.

A. Introduction

A1. The epidemics of HIV and incarceration are closely intertwined. Criminalization of drug use has resulted in large numbers of people with HIV (PLH) cycling through the correctional system each year.[1-3] Indeed, the US has the highest incarceration rate in the world, with 716 per 100,000 of the national population in prison.[4] In addition, HIV prevalence in prisoners is at least four times higher than that among analogous non-incarcerated populations.[5-10] Across the US, incarcerated persons in the Northeast have the highest HIV prevalence (5.3%).[11]

A2. Criminal justice involvement is a risk factor for poor HIV treatment outcomes. Incarceration of PLH has been associated with suboptimal HIV treatment outcomes,[12] particularly in cases of short-term jail detentions,[13,14] and is often disruptive to continuity of HIV care. Studies show that PLH incarcerated within 12 months of initiating antiretroviral therapy (ART) in the community have a higher probability of non-adherence and consequent virologic failure after release than those who are not incarcerated.[15] Among PLH who inject drugs in Baltimore, over 50% of those who were incarcerated experienced virologic failure after a median time of only 6 months, which was double the failure rate seen in those who were not incarcerated.[14]

Repetitive criminal justice system (CJS) involvement, often referred to as the cycle of recidivism, negatively impacts individuals' biological outcomes[16,17] and increases HIV-related mortality risk[13] by interfering with viral suppression. The extent of one's exposure to the CJS seems to have a dose-dependent effect on the post-release likelihood of poor linkage to HIV-related care,[14] non-adherence to ART,[18] and virologic failure.[14] This is consistent with the current theory that recidivists experience worse HIV treatment outcomes after release than non-recidivists,[19] although a controlled comparison has never been undertaken. In addition, CJS-involved women and racial/ethnic minorities appear to be at particularly high risk for HIV infection and poor HIV treatment outcomes.[20,21]

A3. Incarceration can also function as an opportunity to diagnose HIV and initiate treatment. HIV testing and treatment are now available in many correctional settings. As prisons provide a

highly structured setting in which to diagnose and initiate treatment for HIV,[22,23] CJS involvement can be an opportunity for facilitating retention in HIV care.[17,22] Studies from our group and others within the last decade have shown that PLH generally have well-managed disease during incarceration and that it is the period of time immediately after their release during which they are prone to experiencing poor HIV treatment outcomes; virologic failure is common post-release but often only identified and addressed upon reincarceration.[16,24] A 2004 retrospective cohort study showed that, of 292 PLH who were treated for HIV while in prison, released, and subsequently reincarcerated, 59% achieved viral suppression prior to release.[24] However, upon re-entering the CT prison system after spending ≥3 months in the community, any benefit from ART that was observed during incarceration had been lost.[24] This suggests that it may be release into the community, rather than incarceration itself, that makes individuals vulnerable to poor HIV treatment outcomes. These DOC-based studies, however, were limited in that they could only follow PLH while they were incarcerated; assumptions had to be made, especially about non-recidivists, regarding the success of their HIV treatment during their time in the community.

A4. The longitudinal impact of incarceration and community re-entry on inmates' continuity of HIV care remains poorly understood. Successful treatment of HIV requires immediate linkage to HIV care after release and regular (every 3-6 months) monitoring by HIV-1 RNA viral load (VL).[25] Correctional facilities generally provide each inmate with a 10- to 30-day ART supply at their time of release, under the assumption that inmates will receive an ART refill upon linking themselves to a healthcare provider post-release.[22] In a national study, former jail detainees who met with an HIV provider within 30 days of release had a higher probability of achieving viral suppression within 6 months.[26] In one Texas county, however, only 20% of recently released inmates enrolled in an HIV clinic,[27] and only 17.7% filled ART prescriptions[28] within this 30-day window.

Despite the need to better understand HIV management after release, no observational study to date has effectively collected statewide, longitudinal data on HIV outcomes for PLH during and after their transition from a correctional facility into the community. Research on this topic has been primarily limited to cross-sectional and community-based cohort studies, many of which have recall bias, limited follow-up time, and suboptimal study participant retention rates (typically 50%, with missing data either ignored or imputed). The previously described Yale University DOC-based studies have the benefit of using documented custody and biological data, thus minimizing recall bias, but they have lacked access to post-release disease monitoring data until now.

A5. The effectiveness of transitional linkage-to-care services aimed to improve HIV treatment outcomes post-release is also unclear. Qualitative and mixed-methods studies suggest that poor prison discharge planning can lead to poor linkage to care upon community re-entry.[29] Individuals often have difficulty accessing services in order to meet their basic needs, such as housing and employment, and may relapse to substance abuse or engage in transactional sex work soon after their release.[30] These behaviors increase their risk of transmitting HIV, being reincarcerated,[30] and, likely, defaulting from ART. In some US states, transitional linkage-to-care (TLC) and/or case management programs have been implemented to help connect inmates to the healthcare, substance abuse treatment, mental health, housing, and vocational services they require upon release.[31]

Studies on the effectiveness of such programs have primarily followed former jail detainees in the community for ≤6 months and have found mixed results. Most of these studies have been based in the EnhanceLink Initiative, a national project that evaluated TLC interventions at ten US jail sites.[31] Despite using TLC services, only 58% of jail detainees visited an HIV clinic within the first 3 months post-release and only 47% had a visit in the second 3-month period.[32] In contrast, the only known study that has been conducted in prison settings showed high care retention rates irrespective of TLC services.[33] Thus, TLC services appear to be beneficial, at least in jail settings,[29] but more research is needed order to appropriately target and manage the needs of inmates most at risk for poor HIV treatment outcomes.

A6. Connecticut is an ideal setting for exploring the longitudinal impact of incarceration on HIV treatment outcomes. Consistent with the majority of the country, substance abuse in CT is a major contributor to both HIV infection[34] and incarceration.[35] In 2012-2013, CT's incarceration rate for adult US residents averaged 431 per 100,000.[36] In addition, CT has one of the highest recidivism rates nationally; of inmates released in 2004, 43.7% were reincarcerated within 3 years.[37] The general population in CT has an HIV prevalence of 0.36%, which ranks 11th among all US states and 2nd among the six US states with integrated correctional health systems.[11,38] While at least 1.6% of male inmates are known to the DOC as having HIV, the prevalence rate is more than twice as high (3.9%) among female inmates.[11] In addition, blacks and Hispanics disproportionately comprise 24% of the state population but approximately 65% of all PLH in CT.[34]

In the previously mentioned prisoner cohort studies conducted at Yale University, which used CT DOC data similar to data we are proposing to use in this study, it was discovered that only half of PLH who were virally suppressed prior to release were still suppressed upon reincarceration.[19,24] While only one-third of prison entrants were virally suppressed at time of incarceration, ART regimens, after being optimized, became effective for 70% of inmates during their incarceration period.[23] In addition, disparities were noted in that HIV-infected CT inmates were more likely than members of the general CT prison population to be female, of ethnic or racial minorities, and substance abusers.[24] Yet blacks and Hispanics were less likely than whites to be virally suppressed upon entry to the DOC, and women who had achieved viral suppression during a prior incarceration were less likely than men to be observed upon reincarceration to have maintained suppression.[39]

The timeline of HIV-related events and the combination of factors that ultimately lead to these poor outcomes being observed upon reincarceration remain unclear. Linking correctional data to community-based clinical data has recently been recognized as an innovative but infrequently used strategy to address unanswered questions regarding longitudinal HIV outcomes in CJS-involved populations.[40] In addition to having access to and experience with linking correctional databases based on inmate ID number,[19,23,39] Drs. Altice and Meyer at Yale University recently acquired access to longitudinal, community-based HIV surveillance data from the CT Department of Public Health (DPH). Thus, the goal of the proposed study is to use these data to follow CJS-involved PLH longitudinally after release into the community in order to gain a better understanding of the timing of linkage to and retention in HIV care and rates of viral suppression in this vulnerable population.

A7. Innovation

The majority of inmates experience virologic failure after release into the community. While this is presumably due to insufficient engagement in HIV care, the timing of care engagement and treatment failure remains poorly understood. Previous studies have been limited to examining HIV treatment outcomes in specific sub-populations of PLH (e.g., drug users) either during incarceration or after release. In contrast, the proposed study will allow all CJS-involved PLH to be followed longitudinally in the community, regardless of whether they re-enter the CJS at a later date. This study will be the first to link data from an integrated DOC and a DPH HIV surveillance system at a statewide level, and is highly innovative in its ability to: **1)** assess the likelihood and timing of post-release linkage to HIV care, retention in care, and virologic failure for all CJS-involved PLH, and **2)** evaluate the effects of factors such as TLC services utilization, viral suppression at time of release, and the full complexity of one's interaction with the CJS (e.g., length and frequency of incarceration, year of release, and correctional supervision in the community by means of probation or parole) on post-release HIV treatment outcomes. Additional strengths include the potential ability to explore sex and racial/ethnic disparities, which is typically limited by small sample size in observational cohort studies.

B. Aims

The innovative combined use of CJS-based DOC data and community-based eHARs data will allow me to objectively assess HIV treatment outcomes for all PLH released from a CT DOC facility during 2007-2015, according to the following specific aims:

Aim 1. Assess post-release linkage to HIV care: (a.) Determine the proportion of PLH with a VL monitored within three critical time windows following release: 1, 3, and 6 months post-release.[25] **(b.)** Among the PLH who are linked to care (as defined above based on VL monitoring), determine the proportion that demonstrate virologic failure (VL>400 copies/mL[25]) at any point during these time windows. **(c.)** Estimate the median time to first VL post-release.

Aim 2. Assess post-release retention in care, using two different accepted definitions of retention: (a.) Examine the proportion of PLH with ≥2 VLs drawn at least 3 months apart during the first 12 months post-release and, among the PLH who fulfill this definition of retention in care**,** the proportion that demonstrate virologic failure during these 12 months. **(b.)** For PLH who are linked to care within 3-6 months after release (per Aim 1a. criteria), examine the proportion of PLH with a VL monitored within 3-6 months after the initial linkage-to-care VL. Additionally, among the PLH who fulfill this definition of retention in care, examine the proportion that demonstrate virologic failure during these time frames. **(c.)** Estimate the median length of time to the next VL that is drawn after the first post-release VL.

C. Methodology

C.1. Study population: The subjects of interest are PLH in CT with a history of involvement in the CT DOC, an integrated correctional system composed of 17 facilities (16 for men, 1 for women) for both pre-trial jail detainees and sentenced prisoners. Of the 16,594 individuals (both HIV-infected and uninfected) incarcerated in 2014, 94% were men, and 41% self-identified as non-Hispanic black, 31% as non-Hispanic white, and 26% as Hispanic.[41] By 2010 year-end estimates, at least 1.7% of persons in CT DOC custody had diagnosed HIV (1.6% of men, 3.9% of women).[11] This is likely an underestimate of the true HIV prevalence due to under-reporting, under-diagnosis, and non-disclosure of HIV status. In addition, CT DOC data spanning the years 2005-2012 demonstrate that the number of women and racial/ethnic minorities in the CJS with diagnosed HIV is disproportionately higher that the number of minorities in the general CJS population (19.1% of incarcerated PLH are female, 47.5% non-Hispanic black, and 32.3% Hispanic).[23]

C.2. Data sources, database linkage, and selection of study participants from existing data:

C.2.1. Description of the CT DOC database: Upon intake into the CT DOC, data are collected on individuals' demographic characteristics and medical and psychiatric health, including substance use disorders. The DOC also maintains a pharmacy database of all medications, including ART, prescribed during incarceration and has an extensive custody database, including dates of incarceration and release, transfer within the DOC or to a hospital, criminal charges, whether the person was in pre-trial detention or received a prison sentence, and whether the person was released on parole or probation (i.e., community supervision). For prior studies, Drs. Meyer, Altice, and their colleagues successfully merged these data sources to create a comprehensive database containing demographic, biological, pharmacy, and custody data corresponding to all incarceration periods in CT during 2005-2012.[19,23,39] However, this DOC cohort does not allow us to follow PLH who are released to the community and never re-incarcerated. Thus, we propose to link these data to the extensive statewide database described next.

C.2.2. Description of CT DPH-managed data (eHARS and TLC): In the 1980s, the Centers for Disease Control and Prevention (CDC) established an HIV surveillance program to collect, analyze, and distribute data on HIV based on mandatory disease reporting. By 2008, all states had implemented confidential HIV infection reporting as part of a name-based electronic surveillance system called the enhanced HIV/AIDS Reporting System (eHARS).[42] This DPH-managed HIV surveillance database includes demographic information (i.e., sex, race/ethnicity, and age) for all PLH in CT, and, for individuals who have been incarcerated, their unique inmate ID number.

eHARS also contains HIV/AIDS diagnosis data, including the mode of HIV exposure and initial VL and CD4 count at time of diagnosis. After diagnosis, all results from HIV monitoring tests (VL and AIDS-defining CD4) conducted at any CT laboratory facility are reported to the DPH and entered into eHARS, which allows for continuous monitoring of HIV disease progression over time. Mortality data are also collected and confirmed by cross-referencing against National Death Index and Social Security records. The DPH also documents inmates' utilization of the DPH-funded Transitional Linkage into the Community statewide program (Project TLC). TLC assigns a case manager to follow PLH recently released from the CT DOC for up to 30 days post-release, assisting them with linkages and referrals to community-based medical and social services. TLC data also include dates of clinic visits with HIV providers working at clinics funded by the Ryan White HIV/AIDS program, which is recognized as the most common source of HIV care for CJS-involved PLH.[31]

C.2.3. Database linkage and selection of study participants from existing data: Drs. Meyer and Altice at Yale have already obtained data for all PLH incarcerated in the CT DOC during the period 6/1/1999 –12/31/2012. Four primary sources of CT DOC data were used to compile this database: 1) statewide database of all demographic data (e.g., birth date, sex, race, marital status, education status, medical insurance status upon DOC entry/exit, and presence of medical and psychiatric health issues, including substance abuse); 2) laboratory database with all VL/CD4 counts collected during incarceration; 3) pharmacy database, including all prescription information (e.g., ART); and 4) custody and criminal offense database, including dates and types of offense charges, intra-system transfers, and community supervision data. These databases were merged for analysis based on inmate number, after which all unique identifiers were removed. Through ongoing collaboration with the CT DOC, we will update create an updated database for individuals released from the CT DOC between 01/01/2007-12/31/2015 and link these data to the eHARS data available from CT DPH.

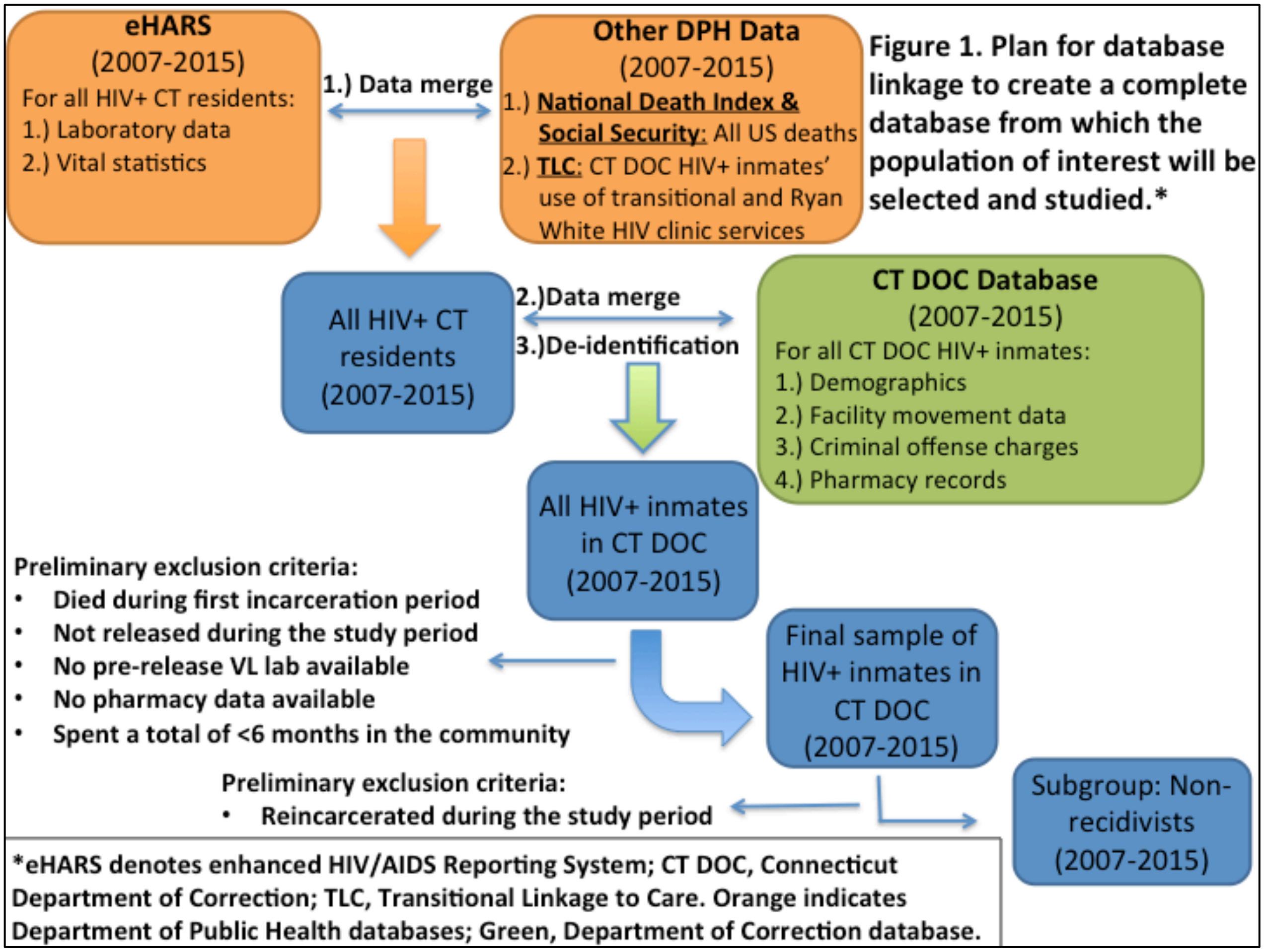

Figure 1. Plan for database linkage to create a complete database from which the population of interest will be selected and studied.*

*eHARS denotes enhanced HIV/AIDS Reporting System; CT DOC, Connecticut Department of Correction; TLC, Transitional Linkage to Care. Orange indicates Department of Public Health databases; Green, Department of Correction database.

As shown in Figure 1, we will work with an experienced data manager at the CT DPH, using SAS 9.4 and the Link Plus probabilistic record linkage program[43] to link CT DOC data with eHARS HIV monitoring, mortality, TLC, and clinic data based on inmate ID number (in accordance with the **Disclosure of Identifiable Health Data Section 19a-25-3(3)**). If necessary, name and date of birth will be used to confirm that the data have been accurately merged. Subsequently, all identifying information including inmate ID number will be replaced with anonymous participant ID numbers prior to analysis. Specifically, the following variables will be requested from eHARS: sex, race/ethnicity, date of first positive HIV test, age at HIV diagnosis, source of HIV report (e.g., private practice, hospital, state facility, federal laboratory, blood bank, etc.), county of HIV diagnosis, disease stage at diagnosis (HIV vs. AIDS), and mode of HIV exposure (e.g., heterosexual, intravenous drug use [IDU], no identified risk factor, or no reported risk). All reported CD4 T-cell and VL values following HIV diagnosis will also be extracted. From the Project TLC database, the types of TLC services provided and the dates of pre- and post-release encounters with TLC case managers will be extracted. Dates of post-release encounters with Ryan White HIV providers will be requested to provide insight into the extent of TLC and HIV care services utilized.

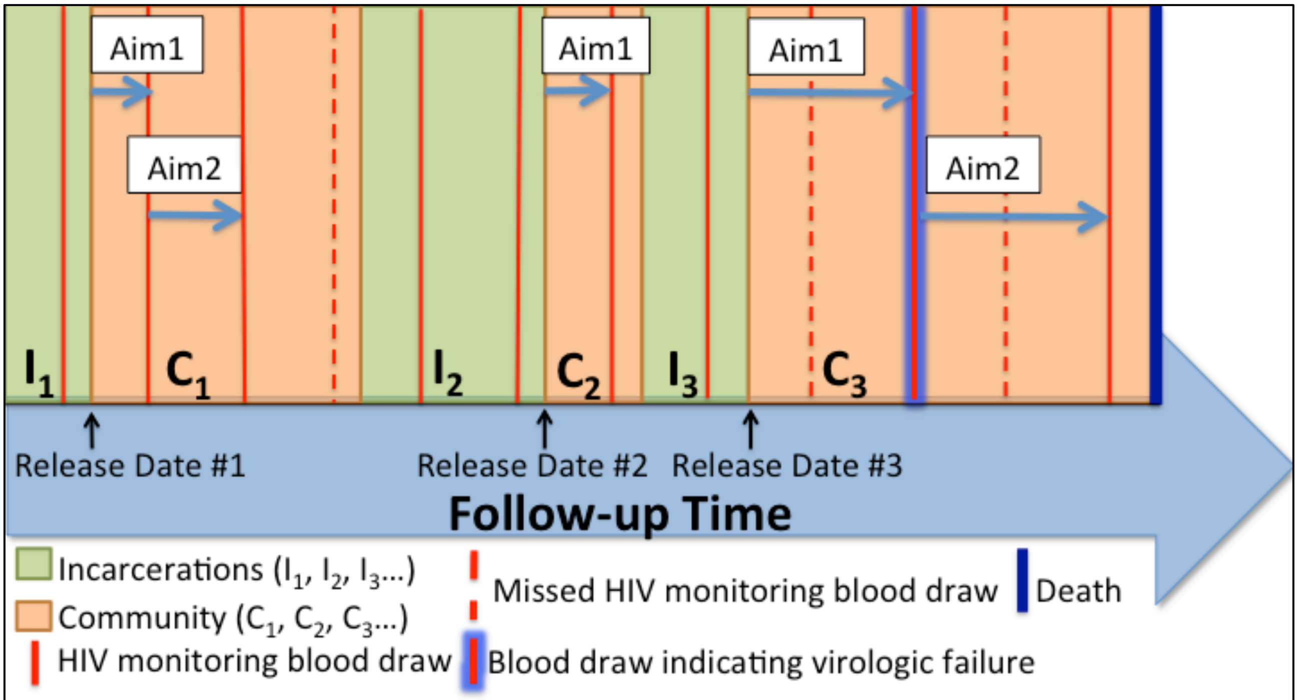

Figure 2. Illustration of the timeline for HIV monitoring laboratory tests for a hypothetical study participant cycling in and out of the correctional system during the study period.

Persons included in the final analysis will meet the following criteria: 1) confirmed HIV seropositive; 2) incarcerated and released at least once from a CT DOC facility during 2007-2015; 3) have at least one set of lab data available during at least one incarceration period; 4) have pharmacy data available during at least one incarceration period; and 5) spend ≥6 months post-release in the community prior to the end of the study period. Based on CT DOC data from 2005-2012, we estimate there will be at least 1,125 eligible subjects released during 2007-2015. After accounting for recidivism rates, we anticipate that these subjects will experience a total of 2,025 incarceration periods with corresponding post-release periods that will be eligible for analysis. We will also conduct a subanalysis limited to non-recidivists (i.e., individuals who are released, spend ≥6 months in the community, and are never reincarcerated during the study period), since data on this subpopulation of CJS-involved PLH are particularly limited in the literature.

C.3. Statistical analysis:

C.3.1. Construction of outcome variables: Throughout the study follow-up period (1/1/2007 – 12/31/2015), each inmate will likely have a series of VL measurements with corresponding test dates. These reported VLs represent times at which individuals interfaced with the healthcare system (both within prison or jail and in the community); because a patient cannot have a VL measured without seeing a provider, VL can be viewed as a proxy for clinic visits and receipt of an ART prescription.

Prisoners receive 30 days of ART post-release and should be evaluated by a provider within this timeframe. In addition, the US Department of Health and Human Services (DHHS) HIV monitoring guidelines dictate that a VL should be repeatedly drawn every 3-4 months for HIV monitoring.[25] For persons on a stable, suppressive ART regimen, the timing between VL monitoring can sometimes be extended to every 6 months.[25,44] Optimal viral suppression of HIV is defined as a VL persistently below the level of detection (which can be as high as 400 copies/mL depending on the assay used).[25]

Our primary study outcomes of interest are: (1) linkage to care, with different degrees of linkage defined as having a VL drawn within 1, 3, or 6 months of release (**Aim 1a**); and (2) retention in care, which will be explored using two different definitions: (**Aim 2a**) having two or more VLs drawn at least 3 months apart within the first year after release, and (**Aim 2b**) having a VL drawn within 3 or 6 months after the first post-release VL. These definitions of linkage to and retention in care imply that the maximum allowable period of time between VLs should generally be 6 months in order to be sufficiently engaged in care in accordance with DHHS HIV monitoring guidelines.[25] A secondary study outcome will be "consistent viral suppression" (<400 copies/mL) during the previously described time frames of interest (**Aims 1b, 2a,** and **2b**). We will also estimate the median length of time between (1) the release date and first post-release VL (**Aim 1c**), and (2) the first and second post-release VLs (**Aim 2c**). These study outcomes will provide insight into how many individuals are sufficiently engaging in HIV care after release as well as the

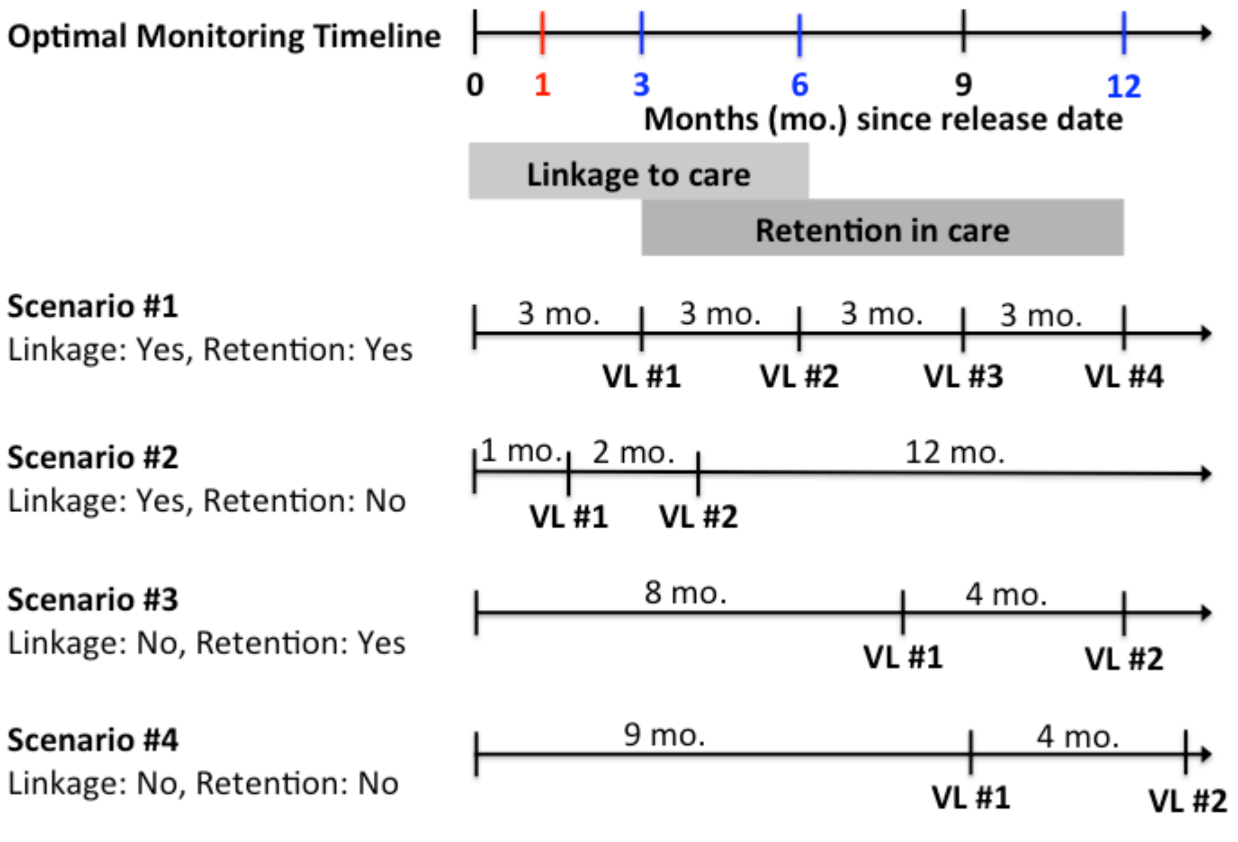

Figure 3. Examples of the practical application of the study definitions of post-release linkage to care and retention in care. *VL denotes viral load.

time points at which individuals most frequently drop out of care and/or develop virologic failure, and the times at which interventions may be most effective at improving these HIV treatment outcomes.

As illustrated in **Figure 3**, each individual's date of release from jail or prison will be used as a start date for the linkage to care and retention in care outcomes. For example, a PLH released on January 1st, 2009, will be evaluated to determine whether a VL was drawn by February 1st, April 1st, and July 1st, 2009 (per **Aim 1a**'s definition of successful linkage to care within 1, 3, and 6 months post-release, respectively). Retention in care will be determined based on whether the individual has at least two reported VLs drawn at least 3 months apart prior to January 1st, 2010 (**Aim 2a**). For **Aim 2b** (not shown in Figure 3), the first post-release VL (e.g., April 1st, 2009) will be used as a start date to determine whether the second post-release VL was drawn within 3-6 months after the first post-release VL (e.g., by July 1st, 2009, or October 1st, 2009). In order to evaluate whether individuals are successfully virally suppressed during these time frames (**Aims 1b, 2a,** and **2b**), all reported continuous VL measurements will be categorized as "viral suppression" or "virologic failure" using an HIV-1 RNA level threshold of 400 copies/mL; if any VL drawn within the time frame under analysis is ≥400 copies/mL, the entire time frame will be coded as corresponding to a virologic failure. Finally, the median time-to-events of interest will be estimated using each individual's: (1) release date (start date) and date corresponding to the first VL drawn in the community (event of interest, **Aim 1c**); and (2) date of first post-release VL (start date) and second post-release VL (event of interest, **Aim 2c**).

C.3.2. Construction of covariates: Covariates will be included in all models to control for potential confounding and to study the exposures of interest alluded to in the Specific Aims (i.e., sex, race/ethnicity, age, history of addiction to a substance, number of health comorbidities, length of incarceration, number of prior incarcerations, supervised release, year of release, viral suppression at time of release, and release with TLC services). Specifically, covariates will include demographic variables such as age at time of release (categorical; 18-34, 35-54, or ≥55 years old), sex (binary), race/ethnicity (categorical; non-Hispanic white, non-Hispanic black, Hispanic, or other), marital status at time of DOC entry (binary; yes/no), education level at time of DOC entry (binary; less than high school vs. completed high school), and medical insurance status upon DOC entry (binary; yes/no) and at time of release (binary; yes/no). Health-specific variables will include HIV transmission route (categorical; heterosexual, homosexual, IDU, or not reported), ART status at time of release (binary; on ART vs. not), viral suppression status at time of release (binary; yes/no, based on whether a VL<400 copies/mL is reported by the DOC within the 3 months leading up to release), a co-morbidity rating (an ordinal 1-5 scale representing the number of non-HIV medical and psychiatric co-morbidities treated while in the DOC), an addiction severity score (an ordinal 1-5 scale representing the extent of one's addiction to a substance on intake, as determined by a trained DOC medical professional), and the extent of TLC services utilized (categorical; none, pre-release only, or post-release). Criminal justice variables will include criminal offense charge data (categorical; based on nature of crime), total number of previous incarcerations in one's lifetime (continuous), length of most recent incarceration (categorical; ≤5 days, 6-30 days, 31-90 days, or >90 days), year of release, and type of community release (categorical; unsupervised, parole, or probation). Because a single individual may experience more than one incarceration and consequently may have more than one post-release period over the course of the study (**Figure 2**), a "release number" variable will be created representing the sequential release period under analysis (categorical; e.g., an individual with three incarcerations during the study period may have up to three different post-release periods which will be chronologically assigned indicator values ranging from 1-3). A continuous "cumulative DOC time" variable will also be created based on the summed length of time each subject has spent in a correctional facility prior to the release period under analysis.

C.3.3. Overview of planned analyses: For the subgroup analysis of PLH who are not reincarcerated during the study period and therefore have only one post-release period to analyze, standard logistic regression and Cox proportional hazards models can be used to examine the

binary endpoints and the time-to-event endpoints of interest, respectively. Otherwise, a generalized estimating equations (GEE) model for binary data[45-47] and a modified Cox proportional hazards model known as the proportional hazards frailty model[48,49] will be used to analyze events of interest after release from each incarceration. These methods will allow us to account for the correlation (ρ) between a single subject's multiple post-release periods where it is possible for the event of interest to occur once during each period. For example, an individual incarcerated three times during the study period may have as many as three post-release periods during which he/she can be followed in the community with regard to post-release HIV treatment outcomes. For individuals with multiple release periods, each release period will be included as an observation and intra-subject correlation will be accounted for by treating each individual as a cluster.

For logistic regression and GEE models, individuals with at least one reported VL during the post-release time period of interest will be assigned a "linkage to care" outcome = 1, whereas individuals lacking a reported VL will be assigned an outcome = 0. Similarly, individuals fulfilling the two different definitions of retention in care will be assigned a "retention in care #1" outcome = 1 (if not fulfilled, outcome = 0) and "retention in care #2" outcome = 1 (if not fulfilled, outcome = 0). The secondary outcome of interest (virologic failure) will only be assessed for the subset of individuals who have a reported VL that can be evaluated; individuals with at least one reported VL≥400 copies/mL within the time frame of interest will be assigned a "virologic failure" outcome = 1, whereas individuals whose VLs are all <400 copies/mL within the time frame of interest will be assigned a "virologic failure" outcome = 0. Outcomes will be examined for each release period (*j*), clustered by individual (*i*). The most robust model will be selected based on minimization of the quasi-likelihood under the independence model criterion (QIC) for GEE.[50]

Similarly, the proportional hazards frailty model for recurrent outcomes will be used to assess the timing of post-release VLs (per **Aims 1c** and **2c**). Individuals not experiencing the outcome of interest during their follow-up time in the community will be right-censored on study end date (12/31/2015), date of reincarceration if applicable, or date of death if prior to the study end date. A model will be selected using minimization of the Bayesian Information Criterion (BIC).[51]

For all study aims, descriptive analyses will first be conducted. Then, for the entire sample (≥1,125 estimated subjects with an anticipated total of 2,025 incarceration periods), GEE modeling will be used to examine the variables associated with VL monitoring within the previously described post-release time frames of interest (**Aim 1a, 2a, 2b**). For the group of individuals who have VLs reported within these time frames, potential predictors for having at least one measurement indicating virologic failure will also explored (**Aim 1b, 2a, 2b**). Finally, analyses using proportional hazards frailty models will be conducted to estimate time-to-first-VL **(Aim 1c)** and time between first and second VL **(Aim 2c)**.

C.4. Strengths and limitations: The key strength of this study lies in the use of existing data extracted from multiple databases, including an integrated correctional health database and a statewide HIV surveillance database. The innovative construction and use of a comprehensive database containing reliable correctional and public health data will enable all CJS-involved PLH in CT to be followed objectively in the community with minimal loss to follow-up. Due to the comprehensive nature of the data, extensive periods of time without a laboratory blood draw will be extremely informative as they will indicate a lack of engagement in HIV care as opposed to poor study retention. Specifically, for inmates who do not have any labs drawn post-release and were never reincarcerated, this study design allows these "missing" VLs to be used to as a study strength, as opposed to a limitation, because the lack of data indicates that these individuals are not successfully being linked to and retained in care.

This study's major limitation is the granularity of patient-level factors that contribute to poor HIV outcomes, including the DOC's absence of detailed substance use history and treatment data and HIV drug resistance profiles. Nevertheless, addiction severity scores on DOC intake will help determine which individuals are at risk for substance abuse relapse post-release, and available pharmacy, clinical, and biological data will help predict which individuals are adhering to ART. Finally, the challenges inherent to linking multiple databases will be mitigated through collaboration with DPH database managers with extensive experience

managing and linking data. Although these CT-specific findings may not be generalizable to US states with very different DOC HIV policies, this study takes significant steps toward informing much-needed health policy targeting at-risk, CJS-involved PLH. It also lays the groundwork for future studies using eHARS and other community-based data to assess and improve HIV outcomes in CJS-involved PLH and other vulnerable populations.

BIBLIOGRAPHY AND REFERENCES CITED

1. United Nations Office on Drugs and Crime. *HIV prevention, treatment and care in prisons and other closed settings: a comprehensive package of interventions.* Austria: UNAIDS;2013.
2. Spaulding AC, Seals RM, Page MJ, Brzozowski AK, Rhodes W, Hammett TM. HIV/AIDS among inmates of and releasees from US correctional facilities, 2006: declining share of epidemic but persistent public health opportunity. *PloS one.* 2009;4(11):e7558.
3. Mathers BM, Degenhardt L, Phillips B, et al. Global epidemiology of injecting drug use and HIV among people who inject drugs: a systematic review. *Lancet.* 2008;372(9651):1733-1745.
4. Walmsley R. *World Prison Population List* International Centre for Prison Studies;2013.
5. Azbel L, Wickersham JA, Grishaev Y, Dvoryak S, Altice FL. Burden of infectious diseases, substance use disorders, and mental illness among Ukrainian prisoners transitioning to the community. *PLoS One.* 2013;8(3):e59643.
6. Azim T, Bontell I, Strathdee SA. Women, drugs and HIV. *The International journal on drug policy.* 2014.
7. Mohamed HI, Saad ZM, Abd-Elreheem EM, et al. Hepatitis C, hepatitis B and HIV infection among Egyptian prisoners: seroprevalence, risk factors and related chronic liver diseases. *Journal of infection and public health.* 2013;6(3):186-195.
8. Reekie JM, Levy MH, Richards AH, et al. Trends in HIV, hepatitis B and hepatitis C prevalence among Australian prisoners - 2004, 2007, 2010. *The Medical journal of Australia.* 2014;200(5):277-280.
9. Semaille C, Le Strat Y, Chiron E, et al. Prevalence of human immunodeficiency virus and hepatitis C virus among French prison inmates in 2010: a challenge for public health policy. *Euro surveillance : bulletin Europeen sur les maladies transmissibles = European communicable disease bulletin.* 2013;18(28).
10. CDC. HIV Among Incarcerated Populations. 2015; http://www.cdc.gov/hiv/group/correctional.html. Accessed October 18, 2015.
11. Maruschak LM. *HIV in prisons, 2001-2010.* Washington, D.C.: Bureau of Justice Statistics, U.S. Department of Justice;2012.
12. Wang EA, McGinnis KA, Long JB, et al. Incarceration and health outcomes in HIV-infected patients: the impact of substance use, primary care engagement, and antiretroviral adherence. *Am J Addict.* 2015;24(2):178-184.
13. Lim S, Harris TG, Nash D, Lennon MC, Thorpe LE. All-cause, drug-related, and HIV-related mortality risk by trajectories of jail incarceration and homelessness among adults in New York City. *American journal of epidemiology.* 2015;181(4):261-270.
14. Westergaard RP, Kirk GD, Richesson DR, Galai N, Mehta SH. Incarceration predicts virologic failure for HIV-infected injection drug users receiving antiretroviral therapy. *Clinical infectious diseases : an official publication of the Infectious Diseases Society of America.* 2011;53(7):725-731.
15. Palepu A, Tyndall MW, Chan K, Wood E, Montaner JS, Hogg RS. Initiating highly active antiretroviral therapy and continuity of HIV care: the impact of incarceration and prison release on adherence and HIV treatment outcomes. *Antiviral therapy.* 2004;9(5):713-719.
16. Stephenson BL, Wohl DA, Golin CE, Tien HC, Stewart P, Kaplan AH. Effect of release from prison and re-incarceration on the viral loads of HIV-infected individuals. *Public health reports.* 2005;120(1):84-88.
17. Milloy MJ, Montaner JS, Wood E. Incarceration of people living with HIV/AIDS: implications for treatment-as-prevention. *Current HIV/AIDS reports.* 2014;11(3):308-316.
18. Milloy MJ, Kerr T, Buxton J, et al. Dose-response effect of incarceration events on nonadherence to HIV antiretroviral therapy among injection drug users. *The Journal of infectious diseases.* 2011;203(9):1215-1221.

19. Meyer JP, Cepeda J, Springer SA, Wu J, Trestman RL, Altice FL. HIV in people reincarcerated in Connecticut prisons and jails: an observational cohort study. *The lancet. HIV.* 2014;1(2):e77-e84.
20. Farel CE, Parker SD, Muessig KE, et al. Sexuality, sexual practices, and HIV risk among incarcerated African-American women in North Carolina. *Women's health issues : official publication of the Jacobs Institute of Women's Health.* 2013;23(6):e357-364.
21. Stein MS, Spaulding AC, Cunningham M, et al. HIV-positive and in jail: race, risk factors, and prior access to care. *AIDS and behavior.* 2013;17 Suppl 2:S108-117.
22. Douglas F, Gallucci AV, Walker S, Dwyer M. *HIV Care in Correctional Settings.* 2014.
23. Meyer JP, Cepeda J, Wu J, Trestman RL, Altice FL, Springer SA. Optimization of human immunodeficiency virus treatment during incarceration: viral suppression at the prison gate. *JAMA internal medicine.* 2014;174(5):721-729.
24. Springer SA, Pesanti E, Hodges J, Macura T, Doros G, Altice FL. Effectiveness of antiretroviral therapy among HIV-infected prisoners: reincarceration and the lack of sustained benefit after release to the community. *Clinical infectious diseases : an official publication of the Infectious Diseases Society of America.* 2004;38(12):1754-1760.
25. U.S. Department of Health and Human Services. AIDSinfo Clinical Guidelines Portal: Guidelines for the Use of Antiretroviral Agents in HIV-1-Infected Adults and Adolescents. 2015; https://aidsinfo.nih.gov/guidelines/html/1/adult-and-adolescent-arv-guidelines/458/plasma-hiv-1-rna--viral-load--and-cd4-count-monitoring. Accessed February 28, 2015.
26. Spaulding AC, Messina LC, Kim BI, et al. Planning for success predicts virus suppressed: results of a non-controlled, observational study of factors associated with viral suppression among HIV-positive persons following jail release. *AIDS and behavior.* 2013;17 Suppl 2:S203-211.
27. Baillargeon JG, Giordano TP, Harzke AJ, Baillargeon G, Rich JD, Paar DP. Enrollment in outpatient care among newly released prison inmates with HIV infection. *Public health reports.* 2010;125 Suppl 1:64-71.
28. Baillargeon J, Giordano TP, Rich JD, et al. Accessing antiretroviral therapy following release from prison. *Jama.* 2009;301(8):848-857.
29. Booker CA, Flygare CT, Solomon L, et al. Linkage to HIV care for jail detainees: findings from detention to the first 30 days after release. *AIDS Behav.* 2013;17 Suppl 2:S128-136.
30. Luther JB, Reichert ES, Holloway ED, Roth AM, Aalsma MC. An exploration of community reentry needs and services for prisoners: a focus on care to limit return to high-risk behavior. *AIDS patient care and STDs.* 2011;25(8):475-481.
31. Spaulding AC, Booker CA, Freeman SH, et al. Jails, HIV testing, and linkage to care services: an overview of the EnhanceLink initiative. *AIDS and behavior.* 2013;17 Suppl 2:S100-107.
32. Althoff AL, Zelenev A, Meyer JP, et al. Correlates of retention in HIV care after release from jail: results from a multi-site study. *AIDS and behavior.* 2013;17 Suppl 2:S156-170.
33. Wohl DA, Scheyett A, Golin CE, et al. Intensive case management before and after prison release is no more effective than comprehensive pre-release discharge planning in linking HIV-infected prisoners to care: a randomized trial. *AIDS and behavior.* 2011;15(2):356-364.
34. Connecticut Department of Public Health: TB H, STD & Viral Hepatitis Section *Epidemiologic Profile of HIV in Connecticut* Hartford, CT: CT Department of Public Health;2013.
35. Department of Correction. Recidivism. 2012; http://www.ct.gov/doc/cwp/view.asp?a=1492&Q=305970. Accessed February 1, 2015.
36. United States Bureau of Justic Statistics. *Prison Inmates at Midyear.* 2009.
37. PEW Center on the States. *State of Recidivism: The Revolving Door of America's Prisons.* Washington, DC2011.
38. Centers for Disease Control and Prevention. *HIV Surveillance Report: Diagnoses of HIV Infection and AIDS in the United States and Dependent Areas.* 2010.

39. Meyer JP, Cepeda J, Taxman F, Altice FL. Sex-Related Disparities in Criminal Justice and HIV Treatment Outcomes: A Retrospective Cohort Study of HIV-infected Inmates. *American Journal of Public Health: In press.*
40. Montague BT, Rosen DL, Solomon L, et al. Tracking linkage to HIV care for former prisoners: a public health priority. *Virulence.* 2012;3(3):319-324.
41. Connecticut Department of Correction. Population Statistics. 2013; http://www.ct.gov/doc/cwp/view.asp?a=1492&Q=270036&docNav=. Accessed June 21, 2013.
42. CDC. HIV Surveillance Supported by the Division of HIV/AIDS Prevention. 2014; http://www.cdc.gov/hiv/statistics/recommendations/publications.html. Accessed January 23, 2015.
43. Centers for Disease Control and Prevention: National Program of Cancer Registries. Registry Plus Link Plus. http://www.cdc.gov/cancer/npcr/tools/registryplus/lp.htm. Accessed August 31, 2015.
44. Mugavero MJ, Davila JA, Nevin CR, Giordano TP. From access to engagement: measuring retention in outpatient HIV clinical care. *AIDS patient care and STDs.* 2010;24(10):607-613.
45. Hanley JA, Negassa A, Edwardes MD, Forrester JE. Statistical analysis of correlated data using generalized estimating equations: an orientation. *American journal of epidemiology.* 2003;157(4):364-375.
46. Huh D, Flaherty BP, Simoni JM. Optimizing the analysis of adherence interventions using logistic generalized estimating equations. *AIDS and behavior.* 2012;16(2):422-431.
47. Li Y, Wileyto EP, Heitjan DF. Statistical analysis of daily smoking status in smoking cessation clinical trials. *Addiction.* 2011;106(11):2039-2046.
48. Lee EW, Wei LJ, Amato DA, Leurgans S. Cox-Type Regression-Analysis for Large Numbers of Small-Groups of Correlated Failure Time Observations. *Nato Adv Sci I E-App.* 1992;211:237-247.
49. Nosyk B, MacNab YC, Sun H, et al. Proportional hazards frailty models for recurrent methadone maintenance treatment. *American journal of epidemiology.* 2009;170(6):783-792.
50. Pan W. Akaike's information criterion in generalized estimating equations. *Biometrics.* 2001;57(1):120-125.
51. Zianging Fan, Runze Li. Variable Selection for Cox's Proportional Hazards Model and Frailty Model. *The Annals of Statistics.* 2002;30(1):74–99.

Protection of Confidentiality

We will adhere to all federal regulations regarding research involving human subjects. Prior to analysis, all health and criminal justice data will be approved for use by the institutional review boards at Yale University and the Connecticut Department of Public Health (CT DPH), and the Research Advisory Committee at the Connecticut Department of Correction (CT DOC).

Current status of applications for study approval from other institutional IRBs:

Yale HIC (including a prisoner representative) – approved (documentation included)

CT Department of Correction Research Advisory Committee Application – under review

1. Risk of Human Subjects

1.a Human Subjects Involvement, Characteristics, and Design

To be included in this study are all persons living with HIV (PLH) released from a CT jail or prison at least once during 2007-2015. The anticipated number of study subjects is 1,125 with approximately half of subjects experiencing more than once incarceration during the study follow-up period. Individuals included in the final analysis will meet the following criteria: 1) confirmed HIV seropositive; 2) incarcerated at least once within a CT DOC facility; 3) have at least one set of lab data available during incarceration; 4) have pharmacy data available during at least one incarceration period; and 5) able to be followed post-release for ≥6 months. Individuals who die during their first incarceration period or are a missing pre-release (within 30 days prior to release) laboratory blood draw will be excluded. In addition to having HIV, individuals may also have diagnosed or undiagnosed psychiatric illness, substance use disorders, and other medical co-morbidities. All participants will be ≥18 years old, given what is known about the epidemiological distribution of HIV in CT.

1.b Sources of Materials

All data used in this study will originate from pre-existing CT DOC pharmacy records and custody and criminal offense charge data, as well as CT DPH TLC and HIV/AIDS surveillance data (eHARS), originally collected for non-research purposes. We will request that our collaborators at the DOC provide us with updated data spanning January 1, 2007 through December 31, 2015. Using subjects' unique inmate ID numbers, our researcher Kelsey Loeliger will work with our collaborating eHARS data manager at DPH to link this updated DOC data with DPH eHARS and TLC data for all research subjects and producing a fully de-identified database for our research purposes.

1.c Potential Risks

We will not be administering surveys, conducting research-related laboratory blood draws, or directly interacting with study participants in any way. As there are no medical procedures or interventions in this protocol, there are no inherent risks to the study. Because the data used for analysis will be fully de-identified without constructing a codebook that could link personal identifiers and protected health information, a breach of confidentiality is highly unlikely. The measures we will take to minimize this risk to the individual subjects are further detailed below.

2. Adequacy of Protection against Risk

2.a. Recruitment and informed consent

Because this study proposes to use data that have already been collected, informed consent will not be obtained. Nevertheless, our plans for the use of these confidential data will first need to be approved by two separate institutional review boards and a research advisory committee as stated above.

2.b. Protection against risk

All data linkage involving the use of personal identifiers will be conducted internally within the DPH. Personal identifiers will only be accessible to select DPH staff members who have been pre-

approved for access to these personal identifiers under a pre-existing DPH IRB application. After the DOC and DPH data have been merged, all subjects will be assigned a unique study code and unique identifiers will be removed before the merged database is shared with our Yale research team. The de-identified database will be kept on password protected and encrypted computers with secure server backup requiring multifactor authentication logons, maintained by Yale University IT. Data files will be stored on a limited access J drive, which is maintained by our Yale AIDS Program data manager and to which access is restricted by Yale University IT. Only research team members working directly on the study will have passwords to the database and servers. Additionally, all research personnel have completed NIH online training on the Protection of Human Subjects and Good Clinical Practice and/or Yale University Human Subjects Protection Training and HIPAA Privacy and Security Training.

3. Potential Benefits of the Proposed Research and Importance of the Knowledge to be Gained

While the risk to the individual is minimal due to multiple safeguards against loss of confidentiality, the potential benefit is significant. The knowledge to be gained holds the potential to guide considerable public health policy advances regarding the management of HIV in criminal justice populations. These advances are expected to benefit criminal justice-involved PLH in the near future, possibly including the same individuals that we are proposing to study.

4. Collaborating Sites

All collaborating sites have protocols in place that have been approved by their respective institutional review boards in order to protect the use of their privileged information. We have already received approval from the Yale Institutional Review Board, which includes a prisoner representative. Our application to CT DOC Research Advisory Committee is currently under review.

Informed Consent Forms - Not applicable due to the retrospective nature of the study.

Questionnaires - Not applicable due to the retrospective nature of the study.

DATE STUDY PROPOSAL NEEDS TO BE SENT TO FUNDING SOURCE AND LISTING OF ANY FUNDING SOURCE:

NRSA F30 Grant Application currently under review by the National Institute for Drug Abuse (NIDA)

STUDY PERIOD FOR WHICH APPROVAL SOUGHT:

Data spanning 2007-2015, which will be analyzed during 2015-2018.

SIGNATURES:

Jaimie P. Meyer, MD, MS

PRINCIPAL INVESTIGATOR

Frederick Altice, MD, MA

CO-INVESTIGATOR

TELEPHONE NUMBER WHERE PRINCIPAL INVESTIGATOR MAY BE REACHED:

Jaimie Meyer: 860-315-0780 (cell) 203-737-6233 (office)

Frederick Altice: 203-737-2883 (office)

IF AN APPLICATION OR PROPOSAL SUBMITTED FOR FEDERAL OR FOUNDATION FUNDING INDICATES THAT HUMAN SUBJECTS APPROVAL IS PENDING REVIEW, PLEASE LIST THE FOLLOWING:

TYPE OF APPLICATION OR GRANT: NRSA F30 Research Training Grant (under review)
(**fellowship, training, research, etc.)**

AGENCY: NIDA **APPLICATION OR GRANT NO., IF KNOWN:** 1F30DA041247-01

Please submit one electronic copy of all documents in PDF format along with one signed original and three hard copies to:

Kate Winkeler
State of Connecticut
Department of Public Health
410 Capitol Avenue-MS# **13TMR**
P.O. Box 340308
Hartford, CT 06134-0308
kate.winkeler@ct.gov

send email copy of correspondence to
diane.aye@ct.gov

if using overnight courier, please use zip 06106

HUMAN INVESTIGATION COMMITTEE (HIC)

CONFIDENTIALITY PLEDGE

I recognize the importance of maintaining the confidentiality of identifiable health data collected by the Connecticut State Tumor Registry and other units of the Connecticut Department of Public Health (DPH), and of assuring the right to privacy of persons, facilities, and agencies which cooperate with the Registry and the DPH or participate in the DPH's data collection efforts. I also understand that the Registry and DPH are legally obligated to protect the privacy of public health information. I have read the Connecticut State Statute Sec. 19a-25 and Sections 19a-25-1 through 19a-25-4 of the Regulations of CT State Agencies concerning confidentiality of records concerning morbidity and mortality and have been advised that DPH can take necessary action if a breach of confidentiality occurs.

I therefore pledge that I will *NOT* divulge the identity of patients, physicians, facilities, or agencies included in data obtained from the DPH with HIC approval with anyone other than:

- members of the Registry staff or other DPH unit from which the data are obtained or
- persons who are also approved for access to the data by DPH HIC and have also signed a DPH confidentiality pledge

Date: 11/13/2015

Individual Pledging to Maintain Confidentiality — CHAIR, HUMAN INVESTIGATION COMMITTEE

Name Kelsey B Loeliger — Name DIANE D. AYE, MPH, Ph.D.

Title MD-PhD Predoctoral Candidate

Address 123 York St, Crown Towers 10A, New Haven, CT 06511

Signature: *Kelsey Loeliger 11/24/2015*

Individual Pledging to Maintain Confidentiality — CHAIR, HUMAN INVESTIGATION COMMITTEE

Name Jaimie Meyer — Name DIANE D. AYE, MPH, Ph.D.

Title Assistant Professor of Medicine (AIDS)

Address 135 College Street, Suite 323, New Haven, CT 06510-2283

Signature: *Jaimie Meyer 11/24/2015*

SIGNATURES:

Individual Pledging to Maintain Confidentiality

☐ Registrar of Tumor Records
Connecticut Tumor Registry

☐ Other DPH unit Representative ______________________
(specify unit)

Rev. 9/01/11

BIOGRAPHICAL SKETCH

NAME<br>Meyer, Jaimie Paul	POSITION TITLE<br>Assistant Professor of Medicine / Project Principal Investigator

EDUCATION/TRAINING (Begin with baccalaureate or other initial professional education, such as nursing, and include postdoctoral training.)

INSTITUTION AND LOCATION	DEGREE	YEAR(s)	FIELD OF STUDY
Dartmouth College, Hanover, NH	B.A.	1996-2000	Anthropology & Native American Studies
University of Connecticut School of Medicine, Farmington, CT	M.D.	2001-2005	Medicine
Columbia University, New York, NY	Residency	2005-2008	Internal Medicine
Yale School of Medicine, New Haven, CT	Fellowship	2008-2011	Infectious Diseases
Yale Center for Interdisciplinary Research on AIDS, New Haven, CT	Fellowship	2010-2012	Interdisciplinary HIV Prevention
Yale School of Public Health, New Haven, CT	M.S.	2012-2014	Biostatistics & Epidemiology

Positions

1998	Honorary Service Fellow, Costa Rican Humanitarian Foundation
1999	Spanish Medical Interpreter, Boston Children's Hospital
2000-2001	Research Assistant, UCSF Immunogenetics and Transplantation Laboratory
2002	"Medicine as a Profession" Fellow, Soros Open Society Institute
2009-2012	Preclinical Clerkship Tutor, Yale School of Medicine
2009-present	Infectious Disease Clinician, York Correctional Institute for Women
2012-2014	Instructor, Infectious Diseases, Yale School of Medicine
2014-present	Assistant Professor, Infectious Diseases, Yale School of Medicine

Honors

1998	Inductee, Paelaeopitus Senior Leadership Society
2000	Hannah Croasdale Senior Award
2000	Honors in Medical Anthropology, Dartmouth College
2005	American Medical Women's Association Citation
2005	Connecticut State Medical Society Award
2006	John N. Loeb Intern Award
2011	Bristol Myers-Squibb HIV Virology Fellowship Award
2013	Thornton Award for Clinical Research
2014	Patterson Trust Award in Clinical Research
2014	International Women's Health & Gender Working Group Travel Award
2014	NIDA Women & Sex/Gender Differences Junior Investigator Travel Award
2014	NIH Health Disparities Loan Repayment Program Award

Professional Memberships

2013-present	InWomen's Network, NIDA International Program
2013-present	American College of Physicians
2012-present	Affiliated Scientist, Center for Interdisciplinary Research on AIDS, Yale University
2011-present	American Medical Women's Association
2011-present	Connecticut Infectious Disease Society
2009-present	American Society of Addiction Medicine
2008-present	Infectious Disease Society of America
2005-present	American Medical Association

Selected peer-reviewed publications relevant to the current application:
Peer-Reviewed Journals (in chronological order):

1. Azar M, Springer S, **Meyer J,** Altice F. A Systematic Review of the Impact of Alcohol Use Disorders on HIV Treatment Outcomes, Adherence to Antiretroviral Therapy and Health Care Utilization. Drug and Alcohol Dependence 2010, 112: 178–193. *PMID 20705402. PMC2997193.*
2. **Meyer J**, Springer S, Altice F. Substance Abuse, Violence, and HIV in Women: A Literature Review of the SAVA Syndemic. Journal of Women's Health 2011, 20(7). *PMID 21668380. PMC3130513.*
3. Chen N, **Meyer J,** Springer S. Advances in the Prevention of Heterosexual Transmission of HIV/AIDS among Women in the United States. Infectious Disease Reports, Special Issue: Women and Infectious Diseases 2011; 3: e6. *doi: 10.4081/idr.2011.e6. PMID: 23745166. PMC3671603.*

4. Springer S, Spaulding A, **Meyer J,** Altice F. Public Health Implications for Adequate Transitional Care for HIV-Infected Prisoners: Five Essential Components. Clinical Infectious Disease 2011; 53(5): 469-479. *PMID 21844030 PMC3156144.*
5. **Meyer J,** Qiu J, Chen N, Larkin G, Altice F. Emergency Department Use by Released Prisoners with HIV: An Observational Longitudinal Study. PLoS ONE 2012; 7(8): e42416. *PMID: 22879972 PMC3411742.*
6. **Meyer J,** Qiu J, Chen N, Larkin G, Altice F. Frequent Emergency Department Use among Released Prisoners with HIV: Characterization Including a Novel Multimorbidity Index. Academic Emergency Medicine 13 JAN 2013; 20(1):79-88. *PMID: 23570481 PMC3623800.*
7. **Meyer J,** Wickersham J, Fu J, Brown S, Sullivan T, Springer S, Altice F. Partner Violence and Health among HIV-Infected Jail Detainees. International Journal of Prisoner Health 2013; 9(3): 124-141. *PMID: 24376468 PMC3873166.*
8. Althoff A, Zelenev A, **Meyer J**, Fu J, Brown S, Vagenas P, Avery A, Cruzado J, Spaulding A, Altice F. Correlates of Retention in HIV Care after Release from Jail: Results from a Multi-site Study. AIDS and Behavior 2013 Oct;17 Suppl 2:156-70. *PMID: 23161210. PMC3714328.*
9. Chen N, **Meyer J**, Avery A, Draine J, Flanigan T, Lincoln T, Spaulding A, Springer S, Altice F. Adherence to HIV Treatment and Care among Previously Homeless Jail Detainees. AIDS Behav. 2013 Oct;17(8):2654-66. doi: 10.1007/s10461-011-0080-2. *PMID: 22065234. PMC3325326.*
10. Williams C, Kim S, **Meyer J,** Spaulding A, Teixeira P, Avery A, Moore K, Altice F, Simon D, Wickersham J, Murphy-Swallow D, Ouellet L. Gender Differences in Baseline Health, Needs at Release, and Predictors of Care Engagement among HIV-positive Clients Leaving Jail. AIDS and Behavior 2013 Oct;17 Suppl 2:195-202 *PMID: 23314801. PMC3758427.*
11. Chitsaz E, **Meyer JP**, Krishnan A, Springer SA, Marcus R, Zaller N, Jordan AO, Lincoln T, Flanigan TP, Porterfield J, Altice FL. Contribution of Substance Use Disorders on HIV Treatment Outcomes and Antiretroviral Medication Adherence Among HIV-infected Persons Entering Jail. AIDS and Behavior 2013 Oct;17 Suppl 2:118-27. *.PMID: 23673792. PMC3818019.*
12. **Meyer J,** Althoff A, Altice F. Optimizing Care for HIV-infected People who Use Drugs: *Evidence-Based Strategies for Overcoming Healthcare Disparities*. Clinical Infectious Disease 2013 Nov; 57(9):1309-17. Epub 2013 Jun 23. *PMID: 23797288. PMC3792721.*
13. **Meyer J,** Zelenev A, Wickersham J, Williams C, Teixiera P, Altice F. Gender Disparities in HIV Treatment Outcomes Following Release From Jail: Results From a Multicenter Study. American Journal of Public Health. 2014 Mar; 104(3): 343-41. *PMID: 24432878. PMC3953795.*
14. **Meyer J,** Cepeda J, Wu J, Trestman R, Altice F, Springer S. Optimization of HIV Treatment during Incarceration: Viral Suppression at the Prison Gate. JAMA Internal Medicine. 2014 May;174(5):721-9. *PMID: 24687044. PMC4074594.*
15. **Meyer J,** Cepeda J, Springer S, Wu J, Trestman R, Altice F. HIV in Prison's Revolving Door: A Longitudinal Cohort Study of Recidivists with HIV. The Lancet HIV. Early Online Publication, 10 October 2014. *doi:10.1016/S2352-3018(14)70022-0. NIHMS ID: 636511.*

Book Chapters

1. **Meyer J,** Altice F. HIV in Injection and Other Drug Users. Somesh Gupta, Bhushan Kumar, eds. *Sexually Transmitted Infections* 2nd ed. New Delhi, India: Elsevier, 2012: 1061-80. ISBN 978-81-312-2809-8.
2. **Meyer J,** Altice F. Addiction: Transition to the Community. Robert L. Trestman, Kenneth L. Appelbaum, Jeffrey L. Metzner, eds. *Oxford Textbook of Correctional Psychiatry.* Oxford University Press 2014.

BIOGRAPHICAL SKETCH

NAME	POSITION TITLE
Altice, Frederick Lewis	Professor of Medicine, Epidemiology and Public Health / Project Co-Principal Investigator

EDUCATION/TRAINING (Begin with baccalaureate or other initial professional education, such as nursing, and include postdoctoral training.)

INSTITUTION AND LOCATION	DEGREE	YEAR(s)	FIELD OF STUDY
Texas A&M University, College Station, TX	BA	1980	Biology
University of Santiago de Compostela, Spain	MA	1982	Spanish Literature
Emory University School of Medicine, Atlanta, GA	MD	1986	Medicine
Yale University School of Medicine, New Haven, CT	Residency	1986-89	Internal Medicine
Yale University School of Medicine, New Haven, CT	Fellowship	1989-92	Infectious Diseases

Positions and Honors

1991-present	Director, HIV in Prisons Program, AIDS Program, Yale School of Medicine
1992-present	Director, Community Health Care Van, AIDS Program, Yale School of Medicine
1993-1999	Firm Chief, Atkins AIDS Service, Yale-New Haven Hospital
1993-1999	Assistant Professor, Yale School of Medicine
1999 -2008	Associate Professor, Yale School of Medicine
2003-present	Director of Clinical and Community Research, Yale University
2008-present	Professor of Medicine, Yale School of Medicine
2010-present	Icon Professor of Medicine, University of Malaya, Kuala Lumpur, Malaysia
2011-present	Professor of Epidemiology and Public Health

Other Experience and Professional Memberships

1989-present	Fellow, American College of Physicians
1991-present	Member, Infectious Diseases Society of America, American Public Health Association
1994-present	International AIDS Society
1995-present	Society of Correctional Physicians, National Commission on Correctional Health Care
2000-2001	Congressional Commission on Health Status of Soon-to-be-Released Prisoners (ID Chair)
2000 – 2010	Member, National Institutes of Health. NIDA K Career Development Award Scientific Review Group
2005 – present	Member, American Society of Addiction Medicine
2004 – present	AAHIVM-American Academy of HIV Medicine
2006 – 2008	Chair, WHO, Guidelines Committee-Treatment Integration of HIV-TB Among Drug Users
2011 – present	Member, International Association of Physicians in AIDS Care Guidelines Committee on Adherence to Antiretroviral Therapy and Retention in Care (Substance Use Chair)
2013 – present	Member, HPTN Committee, Substance Use & MSM Protocol Team

Selected Publications (from among 248): underlined are current/previous trainees

Recent Publications with Co-investigators:

1. **Loeliger KB**, Marcus R, Wickersham JA, Pillai, V, Kamarulzaman A, Altice FL. The Syndemic of HIV, HIV-related Risk and Multiple Co-morbidities Among Women Who Use Drugs in Malaysia: Important Targets for Intervention. *Addictive Behaviors.* 2015: In Press.
2. Wickersham JA, Loeliger KB, Marcus R, Pillai V, Kamarulzaman A, **Altice, FL.** Correlates of Active and Remote Injection Drug Use Among Women in Malaysia. *American Journal of Drug and Alcohol Abuse*. 2015: In press
3. Al-Darraji HA, Wong KC, Yeow DG, Fu JJ, Loeliger K, Paiji C, Kamarulzaman A, **Altice FL.** Tuberculosis screening in a novel substance abuse treatment center in Malaysia: implications for a comprehensive approach for integrated care. J Subst Abuse Treat. 2014 Feb;46(2):144-9. doi: 10.1016/j.jsat.2013.08.023. Epub 2013 Sep 24. PubMed PMID: 24074846.

Documenting Expertise in Longitudinal Data on Addiction, HIV and Criminal Justice

4. Meyer JP, Cepeda J, Wu J, Trestman RL, Springer SA, **Altice FL.** Optimization of Human Immunodeficiency Virus Treatment During Incarceration: Viral Suppression at the Prison Gate. JAMA internal medicine. 2014;174(5):721-729. PMID: 24687044.
5. Meyer JP, Cepeda J, Springer SA, Wu J, Trestman RL, **Altice FL.** HIV in people reincarcerated in Connecticut prisons and jails: an observational cohort study. *The Lancet. HIV.* 2014;1(2):e77-e84. PMID: 25473651
6. Althoff AL, Zelenev A, Meyer JP, Fu J, Brown SE, Vagenas P, Avery AK, Cruzado-Quiñones J, Spaulding AC, **Altice FL.** Correlates of Retention in HIV Care After Release from Jail: Results from a Multi-site Study. AIDS Behav. 2013;17 Suppl 2:156-170. PMID: 23161210.
7. Morozova O, Azbel L, Grishaev Y, Dvoryak S, Wickersham JA, **Altice FL.** Ukrainian prisoners and community reentry challenges: implications for transitional care. Int J Prisoner Health. 2013;9(1):5-19. PMID: 23527238.

8. Fu JJ, Herme M, Wickersham JA, Zelenev A, Althoff A, Zaller N D, . . . **Altice F L**. Understanding the revolving door: individual and structural-level predictors of recidivism among individuals with HIV leaving jail. *AIDS Behav.* 2013;17 Suppl 2:S145-155. PMID: 4049299
9. Krishnan A, Wickersham JA, Chitsaz E, Springer SA, Jordan AO, Zaller N, **Altice FL**. Post-release substance abuse outcomes among HIV-infected jail detainees: results from a multisite study. *AIDS Behav.* 2013;17 (Suppl 2):S171-180. PMID: 3600070
10. Zelenev A, Marcus R, Kopelev A, et al. Patterns of homelessness and implications for HIV health after release from jail. *AIDS and Behavior.* 2013;17 Suppl 2:S181-194.
11. Springer SA, Qiu J, Saber-Tehrani AS, **Altice FL.** Retention on buprenorphine is associated with high levels of maximal viral suppression among HIV-infected opioid dependent released prisoners. PLoS One. 2012;7(5):e38335. PMID: 22719814
12. Springer SA, Pesanti E, Hodges J, Macura T, Doros G, **Altice FL**. Effectiveness of antiretroviral therapy among HIV-infected prisoners: reincarceration and the lack of sustained benefit after release to the community. *Clinical infectious diseases : an official publication of the Infectious Diseases Society of America.* 2004;38(12):1754-1760.
13. Wickersham JA, Zahari MM, Azar MM, Kamarulzaman A, **Altice FL.** Methadone dose at the time of release from prison significantly influences retention in treatment: Implications from a pilot study of HIV-infected prisoners transitioning to the community in Malaysia. Drug Alcohol Depend. 2013;132(1-2):378-382. PMID: 23414931.

Documenting Expertise in Infectious Diseases and Addiction

14. **Altice FL**, Kamarulzaman A, Soriano VV, Schechter M, Friedland GH. Treatment of medical, psychiatric, and substance-use comorbidities in people infected with HIV who use drugs. Lancet. 2010;376(9738):367-387. PMID: 20650518.
15. Meyer JP, Althoff AL, **Altice FL**. Optimizing Care for HIV-Infected People Who Use Alcohol and Drugs: Evidence-Based Approaches to Overcoming Healthcare Disparities. Clin Infect Dis. 2013;57(9):1309-1317. PMID: 23797288.
16. Thompson MA, Mugavero MJ, Amico KR, Cargill VA, Chang LW, Gross R, Orrell C, **Altice FL** et al. Guidelines for improving entry into and retention in care and antiretroviral adherence for persons with HIV: evidence-based recommendations from an *International Association of Physicians in AIDS Care* panel. Ann Intern Med. 2012;156(11):817-833. PMID: 22393036.

Other researchers (Last, first, middle): Loeliger, Kelsey Burk

BIOGRAPHICAL SKETCH

NAME	POSITION TITLE
Loeliger, Kelsey Burk	Pre-doctoral Researcher / MD-PhD Candidate / Project Investigator

EDUCATION/TRAINING (Begin with baccalaureate or other initial professional education, such as nursing, and include postdoctoral training.)

INSTITUTION AND LOCATION	DEGREE	START DATE	END DATE	FIELD OF STUDY
University of Maryland-Baltimore County (UMBC) (Baltimore, MD)	B.S.	09/2003	05/2008	Biological Sciences, Minor: Philosophy
Yale School of Medicine (New Haven, CT)	M.D.	09/2010	Anticipated 05/2018	Medicine
Yale School of Public Health (New Haven, CT)	Ph.D.	09/2013	Anticipated 05/2017	Epidemiology of Microbial Diseases

Positions and Honors

ACTIVITY/ OCCUPATION	START DATE	END DATE	FIELD	INSTITUTION/ COMPANY	SUPERVISOR/ EMPLOYER
Teaching Fellow	08/15	8/16	Designing a research thesis proposal	Yale School of Medicine, Physician Associate Program	Rosana Gonzalez-Colaso, PharmD, MPH
Teaching Fellow	05/15	08/15	Epidemiology	Yale School of Public Health	Mayur Desai, PhD
Research Affiliate	05/15	05/18	HIV, substance abuse, mental health	Yale Center for Interdisciplinary Research in AIDS (CIRA)	Robert Heimer, PhD Frederick Altice, MD/MA
Researcher	05/14	08/14	HIV, ART adherence	Church of Scotland Hospital, South Africa	Gerald Friedland, MD Anthony Moll, MD
Research Affiliate	05/11	08/11	HIV, substance abuse, women's health	University of Malaya, Center of Excellence for Research on AIDS	Frederick Altice, MD/MA Adeeba Kamarulzaman, MBBS/FRACP
Research Assistant	07/10	08/10	Malaria	Yale School of Medicine	Erol Fikrig, MD
Postbaccalaureate Research Training Fellow (IRTA)	06/09	07/10	Aplastic anemia, myelodysplastic syndromes	National Heart, Lung, & Blood Institute (NIH)	Elaine Sloand, MD Neal Young, MD
Intern	03/09	05/09	Health policy, environmental conservation	U.S. Department of State, Bureau of Oceans, Environment, & Science	Hollis Summers, BA
Adolescent Reading Tutor	09/08	03/09	Education research	Haskins Laboratories / Kennedy Krieger Institute	Angela Katenkamp, BS
Co-instructor & Peer Mentor	05/08	08/08	Education, Math/English	UMBC, Collegiate Summer Institute	Lisa Benjamin, BA
Research Assistant	05/06	05/08	Structural biology, retroviruses	UMBC, Howard Hughes Medical Institute	Michael Summers, PhD
Teaching Assistant	09/04	05/05	Biology Lab	UMBC	Lark Claussen, PhD

<u>Academic and Professional Honors</u>

2014 Infectious Disease Society of America Medical Scholars Award
2014 Yale Downs International Health Student Travel Fellowship
2014 Yale Office of Student Research Lowe Fund
2014 Yale Office of Student Research Summer Research Fellowship
2008 Summa Cum Laude, UMBC

2008 UMBC Honors College
2007 Rohm & Haas Research Merit Award
2003-2008 UMBC Dean's List
2003-2008 UMBC President's List
2003-2008 UMBC University Scholar Award (full undergraduate academic scholarship)

Memberships in Professional and Honor Societies
2014 Infectious Diseases Society of America (IDSA)
2012 American Medical Student Association (AMSA)
2012 American Medical Association (AMA)
2006 Phi Kappa Phi Honors Society (top 7.5% of college juniors)
2005 Phi Beta Kappa Honor Society (considered the most prestigious US academic honor society)
2005 Golden Key International Honor Society (top 15% of college and graduate students)
2004 National Society of Collegiate Scholars (top 20% of first- and second-year college students)

See NCBI Bibliography for full list of published and unpublished work:
http://www.ncbi.nlm.nih.gov/sites/myncbi/1f5g0UKFgzjQj/bibliography/47785766/public/?sort=date&direction=ascending

Recent publications – Epidemiology of HIV, tuberculosis, and substance abuse in Malaysia and South Africa:

1. **Loeliger KB,** Niccolai, LM, Mtungwa, N, Moll, A, Shenoi, S. Community Health Workers Perspectives on Managing HIV/AIDS in Rural South Africa: Interventional Strategies to Improve Enrollment in Antiretroviral Therapy. [Manuscript in preparation for *AIDS Care*]
2. **Loeliger KB,** Niccolai, LM, Mtungwa, N, Moll, A, Shenoi, S. Community Health Worker Perspectives on Barriers to HIV+ Patients' Initiation of and Retention in Antiretroviral Therapy in Rural South Africa. [Manuscript under review by *AIDS Care,* AC-2015-08-0588]
3. **Loeliger KB**, Marcus R, Wickersham JA, Pillai, V, Kamarulzaman A, Altice FL. The Syndemic of HIV, HIV-related Risk and Multiple Co-morbidities Among Women Who Use Drugs in Malaysia: Important Targets for Intervention. *Addict Behav.* 2015: In Press.
4. Wickersham JA, **Loeliger KB**, Marcus R, Pillai V, Kamarulzaman A, Altice FL. Patterns of Substance Use and Correlates of Active and Remote Injection Drug Use Among Women in Malaysia. *American Journal of Drug and Alcohol Abuse.* 2015: In Press.
5. Al-Darraji HA, Wong KC, Yeow DG, Fu JJ, **Loeliger K**, Paiji C, Kamarulzaman A, Altice FL. Tuberculosis screening in a novel substance abuse treatment center in Malaysia: implications for a comprehensive approach for integrated care. *J Subst Abuse Treat.* 2014 Feb;46(2):144-9. doi: 10.1016/j.jsat.2013.08.023. Epub 2013 Sep 24. PubMed PMID: 24074846.

Publications – Basic science research on retroviral and malarial lifecycles:

6. Zhao YO, Kurscheid S, Zhang Y, Liu L, Zhang L, **Loeliger K**, Fikrig E. Enhanced survival of Plasmodium-infected mosquitoes during starvation. *PLoS One.* 2012;7(7):e40556. doi: 10.1371/journal.pone.0040556. Epub 2012 Jul 10. PubMed PMID: 22808193; PubMed Central PMCID: PMC3393683.
7. Miyazaki Y, Irobalieva RN, Tolbert BS, Smalls-Mantey A, Iyalla K, **Loeliger K**, D'Souza V, Khant H, Schmid MF, Garcia EL, Telesnitsky A, Chiu W, Summers MF. Structure of a conserved retroviral RNA packaging element by NMR spectroscopy and cryo-electron tomography. *J Mol Biol.* 2010 Dec 17;404(5):751-72. doi: 10.1016/j.jmb.2010.09.009. Epub 2010 Oct 8. PubMed PMID: 20933521; PubMed Central PMCID: PMC3023341.
8. Miyazaki Y, Garcia EL, King SR, Iyalla K, **Loeliger K**, Starck P, Syed S, Telesnitsky A, Summers MF. An RNA structural switch regulates diploid genome packaging by Moloney murine leukemia virus. *J Mol Biol.* 2010 Feb 12;396(1):141-52. doi: 10.1016/j.jmb.2009.11.033. Epub 2009 Nov 17. PubMed PMID: 19931283; PubMed Central PMCID: PMC2836482.

Publications – Translational research on myelodysplastic syndrome and aplastic anemia:

9. Olnes MJ, Shenoy A, Weinstein B, Pfannes L, **Loeliger K**, Tucker Z, Tian X, Kwak M, Wilhelm F, Yong AS, Maric I, Maniar M, Scheinberg P, Groopman J, Young NS, Sloand EM. Directed therapy for patients with myelodysplastic syndromes (MDS) by suppression of cyclin D1 with ON 01910.Na. *Leuk Res.* 2012 Aug;36(8):982-9. doi: 10.1016/j.leukres.2012.04.002. Epub 2012 Apr 21. PubMed PMID: 22524974; PubMed Central PMCID: PMC3381873.
10. Olnes MJ, Poon A, Miranda SJ, Pfannes L, Tucker Z, **Loeliger K**, Padilla-Nash H, Yau YY, Ried T, Leitman SF, Young NS, Sloand EM. Effects of granulocyte-colony-stimulating factor on Monosomy 7 aneuploidy in healthy hematopoietic stem cell and granulocyte donors. *Transfusion.* 2012 Mar;52(3):537-41. doi: 10.1111/j.1537-2995.2011.03313.x. Epub 2011 Aug 29. PubMed PMID: 21883270; PubMed Central PMCID: PMC3235244.
11. Sloand EM, Olnes MJ, Shenoy A, Weinstein B, Boss C, **Loeliger K**, Wu CO, More K, Barrett AJ, Scheinberg P, Young NS. Alemtuzumab treatment of intermediate-1 myelodysplasia patients is associated with sustained improvement in blood counts and cytogenetic remissions. *J Clin Oncol.* 2010 Dec 10;28(35):5166-73. doi: 10.1200/JCO.2010.29.7010. Epub 2010 Nov 1. PubMed PMID: 21041705; PubMed Central PMCID: PMC3020689.

Abstracts and Poster Presentations:

12. **Loeliger KB**, Biggs ML, Seal DW, Gordon MS, Beckwith CG, et al. Gender Differences in Sexual Risk Behaviors Among HIV-infected and Uninfected Persons Involved in the U.S. Criminal Justice System: The STTR Harmonization Project. *National HIV Prevention Conference*; 2015 Dec 6-9; Atlanta, GA. [Upcoming Poster]
13. **Loeliger KB**, Mtungwa N, Niccolai LM, Moll A, Friedland G, et al. Community Beliefs Regarding ART and Barriers to HIV Patients' Initiation and Retention in Care as Perceived by Community Heath Workers in Rural South Africa. *IDWeek*; 2015 Oct 7; San Diego, CA.
14. **Loeliger KB**, Mtungwa N, Niccolai LM, Moll A, Friedland G, et al. Community Beliefs Regarding ART and Barriers to HIV Patients' Initiation and Retention in Care as Perceived by Community Heath Workers in Rural South Africa. *7th South Africa AIDS Conference*; 2015 June 12; Durban, South Africa.
15. Wickersham JA, **Loeliger KB**, Pillai V, Marcus R, Kamarulzaman A, et al. Drug use and injection risk behaviours among female drug users in Malaysia: Results from a pilot study. *International AIDS Conference*; 2013 June 30; Kuala Lumpur, Malaysia.
16. **Loeliger KB**, Iyalla K, Miyazaki Y, Summers MF. Dimerization of Entire Psi-Site of Moloney Murine Leukemia Virus Genome Exposes Multiple Nucleocapsid Protein Binding Sites. *Keystone Symposia on Molecular and Cellular Biology: Frontiers of Structural Biology*; 2008 January 06; Steamboat Springs, CO.

Certificate of Completion

The National Institutes of Health (NIH) Office of Extramural Research certifies that **Jaimie Meyer** successfully completed the NIH Web-based training course “Protecting Human Research Participants”.

Date of completion: 12/01/2015

Certification Number: 1929719

Certificate of Completion

The National Institutes of Health (NIH) Office of Extramural Research certifies that **Kelsey Loeliger** successfully completed the NIH Web-based training course “Protecting Human Research Participants”.

Date of completion: 11/24/2015

Certification Number: 1925784

Yale University

Human Investigation Committee
55 College Street
New Haven CT, 06510

Telephone: 203-785-4688
Fax: 203-785-2847
http://info.med.yale.edu/hic

**To:**	Jaimie Meyer, M.D.
**From:**	The Human Investigation Committee
**Date:**	09/15/2015
**HIC Protocol #:**	1106008696
**Study Title:**	Effect of Newer Antiretroviral Regimens on HIV Biological Outcomes in HIV-infected Prisoners: A 13-Year Retrospective Evaluation
**Committee Action**	**Expedited Approval**
**HIC Action Date:**	09/15/2015
**Expiration Date:**	06/29/2016
**Submission Type:**	**Amendment**

This protocol was amended following an expedited review by the Human Investigation Committee. This review meets approval criteria set forth in 45 CFR 46.111. Please be advised that the protocol is due to be reapproved by the expiration date noted above.

Review Comments:

- The amendment to allow for further analyses, with the addition of data from the CT Department of public Health.
  The aims of this modified study are:
  1.)To use the same previously-used custody, criminal justice, pharmacy, and laboratory data collected and maintained by the CT Department of Corrections (DOC), but updated by the CT DOC to include data through 2012 and 2013.
  2.)To merge this data with statewide HIV/AIDS surveillance data available through the enhanced HIV/AIDS Reporting System (eHARS), managed and protected by the CT Department of Health (DPH).

  The amendment is approved in its entirety.

It is the investigator's responsibility to apply for reapproval prior to the Expiration Date noted above. Please allow two months for reapproval.

If you have not already done so, please **prepare a current, comprehensive protocol inclusive of all previously approved amendments by the time of this protocol's renewal.** This means that the changes must be incorporated into the body of the document.
